# The Relationship between Early Maladaptive Schemas and Problematic Facebook Use: The Indirect Effects of Perceived Stress

**DOI:** 10.3390/ijerph20042969

**Published:** 2023-02-08

**Authors:** Andrzej Cudo, Dorota Mącik, Mark D. Griffiths

**Affiliations:** 1 Institute of Psychology, The John Paul II Catholic University of Lublin, Aleje Racławickie 14, 20-950 Lublin, Poland; 2 International Gaming Research Unit, Psychology Department, Nottingham Trent University, Nottingham NG1 4FQ, UK

**Keywords:** early maladaptive schemas, problematic Facebook use, perceived stress, external stress, behavioral addictions

## Abstract

Facebook is one of the most popular social media platforms. As well as facilitating contact and the exchange of information, the use of Facebook can lead to problematic Facebook use (PFU) among a small number of users. Previous studies have shown the relationship between PFU and early maladaptive schemas (EMSs). Additionally, previous studies have reported associations between PFU and perceived stress and between EMSs and perceived stress. Consequently, the main aim of the present study was to investigate the relationship between PFU and EMSs and the role of perceived stress as a mediator of this relationship. The study comprised 993 Facebook users (505 female, M = 27.38 years, SD = 4.79, aged from 18 to 35 years). PFU was assessed using the eight-item Facebook Intrusion Scale, perceived stress was assessed using the Perceived Stress Questionnaire, and EMSs were assessed using the Young Schema Questionnaire (YSQ–S3). The results suggested that insufficient self-control/self-discipline, approval seeking, dependence/incompetence, enmeshment, and entitlement/grandiosity schemas were positively associated with PFU. There was also a negative relationship between PFU and EMSs, such as social isolation/alienation and defectiveness/shame schemas. The findings showed that PFU was positively associated with external stress. Additionally, external stress had an indirect effect in the relationship between mistrust/abuse and PFU, failure to achieve and PFU, and self-punitiveness and PFU. These results contribute to a better understanding of PFU development mechanisms associated with early maladaptive schemas and perceived stress. Additionally, knowing the EMSs associated with PFU and perceived stress might improve the therapeutic interventions and prevention of this problematic behavior.

## 1. Introduction

The development of modern technology has contributed to the expansion of social media platforms, which are defined as *“Internet-based applications, where user-generated content is the lifeblood of social media, individuals and groups create user-specific profiles for a site or app designed and maintained by a social media service, and social media services facilitate the development of social networks online by connecting a profile with those of other individuals and/or groups”* (p. 745, [1]). In 2021, over 4.26 billion individuals used social media worldwide. However, this number is expected to rise to almost 6 billion by 2027 [2]. Consequently, the growing number of social media users raises questions about the consequences of using such platforms. On the one hand, social media can contribute to maintaining contact with friends, followers, and family, the rapid exchange of information in the community, and the possibility of online social support [3]. On the other hand, excessive social media use can contribute to problematic social media use [4,5]. In an analysis of data from 32 countries, Cheng et al. [6] reported that the prevalence of social media addiction was approximately 5% using a monothetic estimation method and approximately 13% using a polythetic estimation method. Here, the monothetic estimation method included individuals who chose a score above 4 (out of 5) on all the Bergen Facebook Addiction Scale (BFAS) [7] or Bergen Social Media Addiction Scale (BSMAS) [8] items. The polythetic estimation method included individuals who scored equal to or above 4 (out of 5) on at least four of the six BFAS or BSMAS items. Consequently, it is important to identify potential factors contributing to the negative consequences of social media use.

### 1.1. Problematic Facebook Use

Despite the development of other social network platforms (e.g., Twitter, TikTok, Instagram), Facebook is still the most popular social network platform worldwide as assessed by the number of monthly active users [9]. Consequently, the present study focused on this social media platform. The negative consequences of Facebook use in its most extreme forms have been described as (among others) ‘Facebook addiction’ [10], ‘Facebook intrusion’ [11], and problematic Facebook use (PFU) [12]. PFU may also be characterized as failing to regulate Facebook use, leading to negative personal consequences [13]. Considering Griffiths’ component model of addiction [14,15,16], the endorsement of all six of the following symptoms would indicate an individual being addicted to Facebook: salience (i.e., Facebook becomes the most important activity in the individual’s life), mood modification (i.e., using Facebook to alter moods), tolerance (i.e., increasing amounts of Facebook use are required to achieve the former mood-modifying effects), withdrawal (i.e., experiencing negative feelings when unable to engage in Facebook use), conflict (i.e., interpersonal and intrapsychic problems caused by excessive Facebook use including the compromising of education, occupation and/or relationships), and relapse (i.e., a return to the previous pattern of Facebook use after an ineffective control of Facebook use) [14,15,16].

Just to reiterate, Griffiths [16] asserts that in order to recognize behaviors such as Facebook use as an addiction, all six listed aforementioned consequences must occur. However, problematic behavior may still exist even when some consequences are absent [16]. It also should be noted that there is no official diagnosis of PFU in any diagnostic manuals. Previous studies have reported the PFU prevalence rate to be approximately 4% [17,18], but none of these studies have used large-scale representative samples. Additionally, female users appear to be more vulnerable to PFU than male users [12,18,19].

Considering the Interaction of the Person-Affect-Cognition-Execution (I-PACE) model [20,21], PFU may be treated as a subtype of addictive behavior. Based on the I-PACE model, the interaction between general and behavior-specific predisposing variables may lead to PFU development. According to the I-PACE model [20,21], the general predisposing variables include temperamental features, genetics, psychopathology, general coping style, and early childhood experiences. Behavior-specific predisposing variables include specific needs, motives, and values (see [20,21]). The interaction between general and behavior-specific predisposing variables may determine the perception and reaction to external and internal triggers associated with PFU.

According to the I-PACE model, it can be assumed that the initial fascination with Facebook and the gratification associated with using it can develop into using Facebook to compensate unsatisfied needs. In this context, the development of PFU may lead the user to use Facebook to meet unsatisfied needs, cope with stressful situations, and negative emotional states. Moreover, negative early childhood experiences (i.e., emotional abuse, physical abuse, trauma, social isolation), inadequate parental styles, negative familial atmospheres, and parents’ own excessive media use may contribute to making Facebook users more vulnerable to the development of addictive behavior and poorer coping with stressful situations in later developmental stages.

Alvarez-Monjaras et al. [22] described the developmental model of addictions, in which they indicated the importance of attachment experiences, mental representations of attachment, and parent–infant interactions in the development of addiction. In this context, early negative experiences (e.g., trauma, negligence, abuse) may lead to the displacement of social cues for addiction-related cues. Consequently, according to the I-PACE model and the developmental model of addictions, it appears that negative early childhood experiences may contribute to an increased likelihood of developing addiction as well as poorer coping with stressful situations in later developmental stages. Therefore, it is important to verify the relationship between early maladaptive schemas (EMSs), which form in reaction to the more negative aspects during childhood development, and PFU and the role of perceived stress as a mediator of this relationship.

### 1.2. Early Maladaptive Schemas and Stress

One of the factors which may explain individuals’ behavior is early maladaptive schemas (EMSs), described by Young as pervasive self-defeating and/or dysfunctional patterns of memories, emotions, and physical sensations, which are elaborated and strengthened throughout an individual’s lifetime [23]. Moreover, Young et al. [21] broadened Beck’s concepts [24,25] and developed schema theory to emphasize the importance of early childhood negative experiences in the development of maladaptive beliefs. According to Young [23], schemas develop in childhood when a child’s core needs (e.g., acceptance, unconditional love, realistic borders) are unmet. Young et al. [23] described some situations connected to neglecting or rejecting a child’s needs: experiencing different kinds of trauma, lack of positive reactions to their needs, overprotectiveness, and internalization of thinking and behavior of significant individuals [26]. Consequently, the child starts to think negatively about themselves and the world. For example, parents may reject the need for autonomy by helping and doing everything for the child. It may lead to the appearance of helplessness (*I cannot cope effectively*) and negative emotions (*I am sad/anxious/angry about it*). When repeated often, it becomes the form of a belief about the self or the world. EMSs tend to be stable during life, despite the evidence that they are false. Currently, 18 EMSs grouped into four or five dimensions have been described [27,28,29]. However, it should be noted that some analyses have not confirmed such structures [30]. The present study implemented the four dimensions proposed by Bach et al. [28] (see Table 1).

Schemas are usually activated in situations connected to the main theme of the schema. The stronger schemas are, the more situations are perceived as stressful. Stress is often understood as a transaction between the environment and the individual. Additionally, stress has been defined as “*a negative emotional experience accompanied by predictable biochemical, physiological, cognitive, and behavioral changes that are directed either toward altering the stressful event or accommodating to its effects*” (p. 653, [31]). Different stimuli become stressors when the individual interprets the situation as threatening or dangerous (primary appraisal) and evaluates their resources or ability to cope as not sufficient (secondary appraisal) [32]. Schemas seem to be connected to both appraisal types. Negative beliefs lead to perceiving different situations as potentially hurtful because of weak self and self-esteem. At the same time, negative beliefs result in a weakening ability to effectively cope [23]. Consequently, the individual feels stressed.

In the case of stress, three dimensions can be distinguished: (i) external stress, (ii) intrapsychic stress, and (iii) emotional tension [33,34]. The most common type is external stress related to situations in the environment. Different strategies taken to deal with demands may be more or less effective. If coping is not successful, individuals may negatively evaluate themselves. Negative assessment, as well as negative thinking, beliefs, judgments, and other mental states, are the sources of the second type (i.e., intrapsychic stress). The third stress dimension is emotional tension, understood as a feeling of anxiety and excessive nervousness, being tired, exhausted, and without motivation for activity [33].

Relationships between stress and EMSs are close. Schemas may intensify all three stress types because of their maladaptive nature [35]. Individuals being aware of their own negative beliefs can be a stressful situation [36]. However, EMSs modify the perception of external situations to more difficult ones. In this context, Alba and Calvete’s [37] longitudinal study showed that the number of social stressors was positively predicted by EMSs included in the disconnection/rejection dimension (abandonment, mistrust/abuse, emotional deprivation, and defectiveness/shame). Additionally, research has confirmed the role of EMSs as a reason for anxiety [38], depression [39], and emotional dysregulation [40]. However, negative emotions are difficult to cope with. Consequently, the individual tries to find their way of coping—not with the situation, but with negative feelings.

Previous research has confirmed the relationship of EMSs to general addictions [41], alcohol use [42], other substance use [43,44,45], anorexia [46,47], binge eating [48], avoiding behaviors [49], overcompensation [50], compulsive sexual behavior [51], gaming disorder [52], problematic Facebook use [53], problematic smartphone use [54], and other behavioral addictions such as shopping addiction and gambling addiction [55]. In the PFU context, previous research [53] showed that PFU was positively associated with insufficient self-control/self-discipline and approval-seeking schemas. Additionally, PFU has been negatively associated with social isolation/alienation and self-sacrifice schemas.

Taken together, EMSs are important in the development of behavioral addictions such as PFU [53], and may also contribute to the perception and coping with stressful situations in daily life [36,37,56]. In this context, it also should be noted that perceived stress has been positively associated with PFU (see [57,58]).

**Table 1:** Description of early maladaptive schemas [53].

Dimension (Bach, Lockwood and Young, 2018 [28])	Dimension Description	Schemas	Description of Schemas
Disconnection and rejection	The schemas are associated with the general belief that the needs for security, stability, care, and acceptance will never be met.	Emotional deprivation	The belief that emotional needs are not important and will not be met by others.
		Defectiveness/shame	The individual’s belief that there is something wrong with them/that they have some permanent defect in them.
		Mistrust/abuse	The individual’s belief that others can hurt, abuse, cheat, or humiliate them.
		Social isolation/alienation	The belief that the individual is completely different from other individuals and does not belong to society.
		Emotional inhibition	The belief that it is necessary to suppress spontaneous emotions and impulses.
		Pessimism/negativism	The belief that everything will turn out badly.
Impaired autonomy and performance	The schemas are associated with dependence on others, feeling insecure, and suffering from a lack of self-determination.	Dependence/incompetence	The individual’s belief that they need considerable help from others to manage everyday responsibilities.
		Vulnerability to harm or illness	The belief that negative incidents can happen at any time and that they will not be able to prevent them.
		Abandonment	The belief that other individuals will be unable to provide emotional support because they will sooner or later leave.
		Enmeshment	Excessive emotional overinvolvement and closeness with another individual or individuals.
		Failure to achieve	The belief that it is not possible to achieve as much as others due to poorer competence/ability.
		Subjugation	The individual’s belief that they must submit to the will of others, because otherwise they will face some unpleasant consequences.
Excessive responsibility and standards	The schemas are associated with high, often impossible-to-meet expectations and a perfectionist approach to achievements.	Self-sacrifice	A desire to satisfy the others’ needs, so as not to hurt them.
		Unrelenting standards	The individual’s belief that they must meet unrealistic and high standards.
		Self-punitiveness	The belief that every mistake deserves severe punishment.
Impaired limits	The schemas associated with lack of responsibility, unstable self-assessment, an inability to achieve distant goals.	Entitlement/grandiosity	The belief of being someone special who has special privileges and can do and say what they want, whether it is acceptable to others or not.
		Insufficient self-control/ self-discipline	The belief that self-discipline is unimportant.
		Approval seeking	The belief that an individual’s value depends on positive social approval.

### 1.3. Problematic Facebook Use and Stress

According to the I-PACE model [20,21], a lack of adequate strategies to cope with daily stress may contribute to the excessive use of new media (e.g., social media platforms, videogames) as a way of coping with negative, stressful situations. Here, Facebook users who treat Facebook use as a way to cope or escape from offline problems and stressful daily events may be more likely to develop PFU [59,60,61]. Meier et al. [59] noted that escape into Facebook and Facebook procrastination were associated with the selection of enjoyable Facebook content, which provides substitute gratifications. In the view of Meier et al. [59], this enjoyable Facebook content may also distract individuals from negative stimuli and situations that occur in their everyday life.

Previous research [57,58] has shown a positive relationship between PFU and daily stressful experiences was associated with inconveniences or difficulties in daily life (e.g., related to family, health, finances, or study) over a 12-month period. Additionally, Brailovskaia et al. [57] reported that depressive symptoms significantly and positively moderated the positive relationship between PFU and daily stress. Brailovskaia et al. [58] also reported that offline social support moderated the relationship between daily stress and Facebook use intensity, and online social support positively mediated the relationship between Facebook use intensity and PFU. Additionally, a one-year longitudinal study indicated that daily stress positively predicted PFU level [62]. Taken together, previous studies [57,58,62] showed that perceived stress was an important factor related to PFU development. Consequently, it is important to understand the factors that may contribute to increased perceived stress as well as those contributing to PFU. In this context, it can be assumed that ESMs can be important variables that can both directly and indirectly (by modifying perceived stress) be associated with PFU.

### 1.4. Aim of the Study

The present study investigated the relationship between EMSs and PFU, as well as perceived stress as mediators of this relationship. The study’s theoretical base was schema theory [23] and the I-PACE model [20,21]. Previous research [53] reported a positive relationship between the belief that self-discipline is unimportant (insufficient self-control/self-discipline schema), the belief that an individual’s value depends on positive social approval (social approval schema), and PFU. Additionally, PFU was negatively associated with the belief that individuals are completely different from others and do not belong to society (social isolation/alienation schema) and a desire to satisfy others’ needs so as not to hurt them (self-sacrifice schema). Consequently, it was hypothesized that there would be a positive relationship between PFU and (i) insufficient self-control/self-discipline schema (H_1_) and (ii) approval-seeking schema (H_2_). Additionally, it was hypothesized that there would be a negative relationship between PFU and (i) social isolation/alienation schema (H_3_) and (ii) self-sacrifice schema (H_4_).

Moreover, previous research has shown that EMSs are associated with PFU [53] and perceived stress [36,37,56]. Alba and Calvete [37] reported a positive relationship between social stressors and EMSs included in the disconnection/rejection dimension (abandonment, mistrust/abuse, emotional deprivation, and defectiveness/shame). The disconnection/rejection dimension has been connected to the general belief that the needs for security, stability, care, and acceptance will never be met [23,28]. Consequently, these beliefs can reinforce the perceived stressfulness of life events. Additionally, previous studies [57,58,62] have reported that perceived stress is positively associated with PFU. Consequently, it can be assumed that Facebook users with schemas from the disconnection and rejection dimension may more easily experience stress in their daily lives, and therefore, be more susceptible to PFU development. Consequently, it was hypothesized that perceived stress dimensions would be positively associated with PFU (H_5_). It was also hypothesized that perceived stress dimensions would mediate the relationship between EMSs included in the disconnection/rejection dimension and PFU (H_6_).

## 2. Materials and Methods

### 2.1. Participants

The study comprised 993 Facebook users (505 female users). The participants’ age ranged from 18 to 35 years (M = 27.38, SD = 4.79). The participants’ characteristics are shown in Table 2. Participants were recruited online from the Polish research panel *Ariadna* and received points for completing the survey. Participants could use these points to receive prizes offered by the *Ariadna* research panel (e.g., cosmetics, electronic devices, games, books). However, it should be noted that the research panel verified each panelist to exclude bots and multiple accounts by one panelist. The study was conducted in accordance with the Declaration of Helsinki, and the first author’s university Ethical Committee approved the study (number KEBN 42/2021). The present study is part of a larger research project examining behavioral addictions funded by the Gambling Problem Solving Fund (Polish: Fundusz Rozwiązywania Problemów Hazardowych), administered by the Minister of Health. Considering the clear focus of the present study, only the variables needed to verify the relationship between PFU, perceived stress, and EMSs were examined. The study was conducted from October 2022 to June 2023. The dataset from the present study is available from the John Paul II Catholic University of Lublin repository database (access link: http://hdl.handle.net/20.500.12153/3890).

### 2.2. Measures

The eight-item Facebook Intrusion Questionnaire (FIQ) [11] (Polish version: [63]; see Supplementary Materials Table S1) was used to assess PFU. Items (e.g., “*I have been unable to reduce my Facebook use*”) are responded to on a seven-point scale, ranging from 1 (*strongly disagree*) to 7 (*strongly agree*), with scores ranging from 8 to 56. Higher scores reflect greater PFU intensity. In the present study, Cronbach’s alpha was 0.92. The FIQ has been used to assess PFU in various countries, including Australia, Cyprus, Greece, Hong Kong, Lithuania, New Zealand, Peru, Poland, Russia, Spain, Turkey, Ukraine, the United Kingdom, and the United States [64].

The 90-item Young Schema Questionnaire (YSQ-S3) [65] (Polish version: [66]) was used to assess 18 EMSs. Individuals answer items using a six-point scale from 1 (*completely untrue of me*) to 6 (*describes me perfectly*). Higher scores reflect a greater intensity of the specific schema. The YSQ-S3 had good psychometric properties in the present study, with Cronbach’s alphas of 0.90 for emotional deprivation, 0.84 for abandonment, 0.86 for mistrust/abuse, 0.88 for social isolation/alienation, 0.92 for defectiveness/shame, 0.89 for failure to achieve, 0.83 for dependence/incompetence, 0.84 for vulnerability to harm or illness, 0.86 for enmeshment, 0.85 for subjugation, 0.75 for self-sacrifice, 0.86 for emotional inhibition, 0.73 for unrelenting standards, 0.69 for entitlement/grandiosity, 0.84 for insufficient self-control/self-discipline, 0.79 for approval seeking, 0.87 for pessimism/negativism, and 0.86 for the self-punitiveness schema.

The 27-item Perceived Stress Questionnaire (PSQ) [34] was used to assess perceived stress in three subscales: (i) external stress (e.g., “*I feel exhausted by constantly proving my point*”), (ii) intrapsychic stress (e.g., “*I have my plans, but I’m afraid I won’t realize them because my psyche is too weak*”), and (iii) emotional tension (e.g., “*I am feeling anxious that more and more things are upsetting me*”). Individuals answer the items using a five-point scale: 1 (*true*), 2 (*somewhat true*), 3 (*neither true or untrue*), 4 (*somewhat untrue*), and 5 (*untrue*). However, it should be noted that the response scale was reversed so that higher scores reflected a greater intensity of the perceived stress in three dimensions. The scale had good psychometric properties in the present study, with Cronbach’s alphas of 0.87 for emotional tension, 0.78 for external stress, and 0.81 for intrapsychic stress.

Participants also answered sociodemographic questions such as age, gender, education level, place of residence, marital status, and the time spent using Facebook per week.

### 2.3. Statistical Analysis

Descriptive statistics were calculated for all variables, such as means and standard deviations. Additionally, the rho Spearman correlation coefficient with a 95% confidence interval was used to explore the relationship between PFU, perceived stress dimensions (emotional tension, external stress, intrapsychic stress), and other analysis variables. The relationships between EMSs, perceived stress dimensions, and PFU were verified using path analysis. Additionally, path analysis was also conducted to examine indirect effects between EMSs and PFU via perceived stress dimensions such as emotional tension, external stress, and intrapsychic stress.

Taking into account previous research reporting a relationship between age, gender, and stress [67,68,69], in order to control these relationships, the relationships between age, gender, and perceived stress dimensions were included in the model. Similarly, the relationships between gender, age, Facebook frequency use, and PFU (see [18,53,62,70,71]) were included in the model. The path model included the covariance between EMSs, gender, and year. Additionally, the covariance between perceived stress dimension residuals was added. However, for clarity, these relationships are presented in Supplementary Materials (Table S2). Considering the multivariate normality assumption violation (Doornik–Hansen omnibus test: χ^2^_(df = 50)_ = 5470.80; *p* < 0.001), the maximum likelihood method with the Sattora–Bentler adjustment [72] was applied. The following fit indices were applied as measures of model fit in the path analysis: χ^2^, SRMR (standardized root mean squared residual), RMSEA (root mean square error of approximation), TLI (Tucker–Lewis Index), and CFI (comparative fit index) [69]. CFI and TLI values higher than 0.90 and RMSEA and SRMR values lower than 0.08 suggest that the model fits the data acceptably [73,74].

The indirect effects were tested using Zhao et al.’s [75] approach comprising the Monte Carlo method (5000 samples) to estimate standardized indirect effects with a 95% confidence interval [76]. The indirect effect was interpreted based on Zhao et al.’s [75] guidelines: (i) *complementary mediation*—indirect effect and direct effect both exist and point in the same direction; (ii) *competitive mediation*—indirect effect and direct effect both exist and point in opposite directions; and (iii) *indirect-only mediation*—indirect effect exists, but no direct effect (full mediation). *IBM SPSS 28* software was used for descriptive statistics and correlation analysis, and *Stata 14* with *medsem.ado* package [76] was used for path analysis.

## 3. Results

### 3.1. Descriptive Statistics and Correlation Analysis Results

The descriptive statistics and correlation analysis results are shown in Table 3. There was a significant positive relationship between PFU and all EMS dimensions and between PFU and perceived stress dimensions. Additionally, the number of hours spent using Facebook per week was positively associated with PFU. Emotional tension, external stress, and intrapsychic stress were positively associated with all EMS dimensions, hours spent using Facebook per week, and gender. There was also a significant negative relationship between age and all perceived stress dimensions.

### 3.2. Path Model Analysis Results

The findings showed the good fitting path model to the data: χ^2^_(df = 3)_ = 4.24; *p* = 0.236; RMSEA = 0.020; SRMR = 0.004; CFI = 0.999; TLI = 0.984. The path analysis showed that PFU was positively associated with dependence/incompetence (β = 0.16; SE = 0.06; *p* = 0.009), enmeshment (β = 0.22; SE = 0.02; *p* < 0.001), entitlement/grandiosity (β = 0.09; SE = 0.04; *p* = 0.018), and hours spent using Facebook per week (β = 0.17; SE = 0.03; *p* < 0.001). There was a positive relationship between the PFU and EMSs such as insufficient self-control/self-discipline (β = 0.10; SE = 0.05; *p* = 0.044) and approval seeking (β = 0.08; SE = 0.04; *p* = 0.039), which supported H_1_ and H_2._ Additionally, there was a significant negative relationship between mistrust/abuse (β = −0.13; SE = 0.06; *p* = 0.040), social isolation/alienation (β = −0.25; SE = 0.05; *p* < 0.001), and PFU, which supported H_3_. However, a negative relationship between self-sacrifice schema and PFU was not found (β = 0.03; SE = 0.04; *p* = 0.463). Therefore, H_4_ was not supported. Path analysis showed that emotional tension was positively associated with abandonment (β = 0.15; SE = 0.05; *p* = 0.006), vulnerability to harm or illness (β = 0.13; SE = 0.06; *p* = 0.039), insufficient self-control/ self-discipline (β = 0.17; SE = 0.05; *p* = 0.001), pessimism/negativism (β = 0.20; SE = 0.06; *p* = 0.002), and gender (β = 0.10; SE = 0.03; *p* < 0.001). Additionally, there was a significant negative relationship between entitlement/grandiosity (β = −0.18; SE = 0.04; *p* < 0.001), self-punitiveness (β = −0.19; SE = 0.05; *p* < 0.001), and emotional tension. Path analysis showed that external stress was positively associated with mistrust/abuse (β = 0.29; SE = 0.06; *p* < 0.001), failure to achieve (β = 0.22; SE = 0.05; *p* < 0.001), subjugation (β = 0.20; SE = 0.06; *p* = 0.001), and gender (β = 0.06; SE = 0.03; *p* = 0.034). Additionally, there was a significant negative relationship between defectiveness/shame (β = −0.16; SE = 0.06; *p* = 0.010), entitlement/grandiosity (β = −0.09; SE = 0.04; *p* = 0.034), self-punitiveness (β = −0.19; SE = 0.05; *p* < 0.001), and external stress. Path analysis showed that intrapsychic stress was positively associated with emotional deprivation (β = 0.14; SE = 0.05; *p* = 0.005), abandonment (β = 0.10; SE = 0.05; *p* = 0.027), failure to achieve (β = 0.23; SE = 0.05; *p* < 0.001), vulnerability to harm or illness (β = 0.19; SE = 0.05; *p* < 0.001), subjugation (β = 0.14; SE = 0.06; *p* = 0.012) and gender (β = 0.10; SE = 0.03; *p* < 0.001). Additionally, there was a significant negative relationship between entitlement/grandiosity (β = 0.19; SE = 0.04; *p* < 0.001), self-punitiveness (β = 0.15; SE = 0.05; *p* = 0.002) and intrapsychic stress. Path analysis showed a positive relationship between external stress and PFU (β = 0.11; SE = 0.05; *p* = 0.016). However, there was nonsignificant relationship between PFU and perceived stress dimensions such as emotional tension (β = 0.09; SE = 0.05; *p* = 0.073) and intrapsychic stress (β = 0.06; SE = 0.05; *p* = 0.239), which partially supported H_5_. Detailed results are shown in Table 4.

### 3.3. Indirect Effect Analysis Results

Indirect effect analysis showed significant indirect effects between specific EMSs and PFU via external stress. More specifically, there was a significant indirect effect between mistrust/abuse and PFU via external stress. Considering Zhao et al.’s [75] framework, the statistically significant direct effect between mistrust/abuse and PFU, and opposite directions between the indirect and direct effect, this result may indicate competitive mediation. Additionally, there was a statistically significant indirect effect between failure to achieve and PFU via external stress. Considering the statistically nonsignificant direct effect between this EMS and PFU, this result may indicate indirect-only mediation (full mediation; Zhao et al., [75]). There was also a significant indirect effect between self-punitiveness and PFU via external stress. Considering the statistically nonsignificant direct effect between this EMS and PFU, this result may indicate indirect-only mediation (full mediation; Zhao et al., [75]). Detailed results are shown in Table 5. However, the indirect effect between EMSs and PFU via emotional tension and between EMSs and PFU via intrapsychic stress was statistically nonsignificant. Consequently, these results partially supported H_6_. The results of indirect effects between EMSs and PFU via emotional tension, and between EMSs and PFU via intrapsychic stress are presented in the Supplementary Materials (Table S3).

## 4. Discussion

The main aim of the present study was to investigate the relationship between PFU and EMSs and the role of perceived stress as a mediator in this relationship. The findings showed that PFU was positively associated with insufficient self-control/self-discipline, approval seeking, dependence/incompetence, enmeshment, and entitlement/grandiosity schemas. There was also a negative relationship between PFU and EMSs, such as social isolation/alienation and mistrust/abuse. The findings showed that PFU was positively associated with external stress dimension. Additionally, external stress had an indirect effect in the relationship between mistrust/abuse and PFU, failure to achieve and PFU, and self-punitiveness and PFU.

### 4.1. The Relationship between the Insufficient Self-Control/Self-Discipline Schema, the Approval-Seeking Schema, and PFU

As hypothesized, there was a positive relationship between PFU and (i) the insufficient self-control/self-discipline schema (H_1_), and (ii) the approval-seeking schema (H_2_). This result suggests that strong beliefs that self-discipline is unimportant (insufficient self-control/self-discipline schema) and that an individual’s value depends on positive social approval (approval seeking) may contribute to increased PFU. Additionally, there was a positive relationship between PFU and a strong belief of being someone special who has special privileges and can do and say what they want, whether it is acceptable to others or not (entitlement/grandiosity schema). It should be noted that these schemas—included in the impaired limits dimension—occur when parents manifest an overprotective parental attitude, in which handicap and a lack of limitations, rules, or boundaries prevail. In this context, parents also display conditional/narcissistic, overprotective, and pessimistic/fearful parenting styles (see [28]). Consequently, the persistence of these schemes in later developmental stages may be related to the lack of responsibility, unstable self-assessment, and inability to achieve distant goals in adulthood. These findings align with previous research on the relationship between EMSs and PFU [53]. Additionally, these results are also in line with previous studies indicating a negative relationship between self-control traits and PFU [71,77,78,79], a positive relationship between narcissism and PFU [70,80,81], and positive relationship between ‘like’-seeking behavior and PFU [82]. Here, the ‘like’-seeking behavior refers to common online behaviors to gain more ‘likes’ from other Facebook users [82].

However, it should be noted that Balcerowska et al. [80] found that PFU was positively associated with a grandiose narcissism dimension, such as admiration demand, and negatively associated with a grandiose narcissism dimension, such as self-sufficiency. The admiration demand reflects the need to be an outstanding person who is noticed, admired by others, and famous [83]. Self-sufficiency reflects the individual’s belief in their individualism, independence, high competence and success, and not being dependent on the approval of others [83]. Consequently, it can be assumed that the narcissistic attitude associated with being admired by others is linked to PFU development. At the same time, the individual’s belief in their superiority is not conducive to developing this problematic behavior. In this context, it should be noted that Bach et al. [28] pointed out two ways in which narcissism develops. In the first way, the family system ignores children’s basic emotional needs, and the most important is meeting their parents’ egoistic needs. In the second way, the child’s needs are paramount to the extent that parents do whatever the child wants and when the child wants it (see [84,85]). Consequently, in the first way, children overcompensate for the lack of satisfaction of emotional needs by creating a grandiose self-image. In contrast, in the second way, children build up the belief that they are superior individuals who are entitled to everything they want (see [84,85]).

In this context, it should be noted that the results of Balcerowska et al.’s [80] study are consistent with the two ways of developing narcissism identified in previous research [28,84,85]. Additionally, it can be assumed that a lack of satisfaction with emotional needs in childhood can lead to a search for admiration and social approval in adulthood. Taken together, it can also be assumed that this EMS formed in childhood may lead to difficulties with delayed gratification, the need for excessive admiration, and approval seeking. However, further research is needed to thoroughly investigate the relationship between EMSs and self-control deficits, narcissism, and approval seeking in the context of PFU development.

### 4.2. The Relationship between the Social Isolation/Alienation Schema and PFU

Additionally, there was a negative relationship between social isolation/alienation schema and PFU, which supported H_3_. The findings were in line with previous research regarding the relationship between EMSs and PFU [53]. Here, the strong belief that individuals are completely different from others and do not belong to society may contribute to decreased problems with Facebook use. In this context, Yoo et al. [86] reported a negative relationship between social isolation/alienation schema and peer connectedness. Moreover, Tang et al. [87] found a positive relationship between online relationships, information online support, and PFU. Considering previous research [86,87] and the EMS framework [65], it can be assumed that individuals who believe themselves as not belonging to society may distance themselves from using popular social media such as Facebook. Consequently, they may be less vulnerable to PFU. However, further research is needed to verify this speculation.

### 4.3. The Relationship between the Self-Sacrifice Schema and PFU

The relationship between the self-sacrifice schema (a desire to satisfy others’ needs so as not to hurt them) and PFU was statistically nonsignificant. Consequently, H_4_ was not supported. However, the results showed a positive relationship between PFU and the belief that an individual is not able to cope with everyday difficulties, make the right decision, or make a good choice, and therefore relies on others (dependence/incompetence schema) and excessive emotional overinvolvement and closeness with other individual or individuals (enmeshment schema). These findings are in line with previous research [88] indicating a positive relationship between psychological vulnerability (“*a pattern of cognitive beliefs reflecting a dependence on achievement or external sources of affirmation for one’s sense of self-worth*” ((p. 120), [89]) and PFU and a negative relationship between social competence and PFU. Additionally, Verseillié et al. [90] reported that PFU was positively associated with cluster B personality traits (characterized by dramatic, overly emotional, or impulsive thinking or behavior) and cluster C personality traits (characterized by anxious, fearful thinking or behavior) [91]. Here, it should be noted that cluster C includes avoidant personality disorder, dependent personality disorder, and obsessive–compulsive personality disorder [91]. The present study’s results suggest that individuals who believe they lack their own social competence and are easily dependent on the opinion and decisions of others may be more vulnerable to PFU.

### 4.4. The Relationship between Perceived Stress and PFU

As hypothesized, there was a positive relationship between perceived stress and PFU. However, there was only a statistically significant relationship between external stress and PFU. Consequently, H_5_ was partially supported. External stress is characterized as the feeling of (i) being unfairly judged by others in different social contexts (at home, at work) and (ii) increasing exhaustion by individuals in defending their own point of view (individuals’ own reasons) in looking at different issues. Additionally, external stress is associated with (i) the feeling of frustration and exhaustion that the expectations and tasks set by others exceed an individual’s own resources, abilities, possibilities to fulfil them, and (ii) experiencing distress and helplessness resulting from a sense of being exploited by others [34].

Consequently, external stress is linked to the individual’s social functioning. These results are in line with previous research indicating a positive relationship between perceived stress and PFU [57,58,62] and a positive relationship between social anxiety and PFU [19,92]. Here, it is reasonable to assume that individuals for whom external social contact factors are stressors may be more vulnerable to PFU development. Moreover, according to the I-PACE model [20,21], a lack of adequate strategies to cope with daily stress associated with social situations may contribute to excessive Facebook use to cope with these negative and stressful situations.

### 4.5. The Relationship between EMSs Included in the Disconnection/Rejection Dimension and PFU via Perceived Stress

The findings showed positive indirect effects between mistrust/abuse (the belief that other individuals are hurting and exploiting, threatening) and PFU via external stress. Additionally, external stress exerted a positive indirect effect on the relationship between failure to achieve (the belief that it is impossible to achieve as much as others due to poorer competence/ability) and PFU. There was also a negative indirect effect between self-punitiveness (the belief that every mistake deserves severe punishment) and PFU via external stress. Consequently, these results partially supported H_6_. More specifically, only the mistrust/abuse schema is part of the disconnection and rejection dimension. In the context of mistrust/abuse schema, there were negative direct effects between the mistrust/abuse schema and PFU, and positive indirect effects between the mistrust/abuse schema and PFU via external stress. Consequently, it can be posited that two mechanisms may exist that are related to these relationships. The first mechanism may be related to the stress of interactions with other individuals. In this context, Sheldon [93] showed that individuals with anxiety and fears in their face-to-face communication might use Facebook to pass the time and feel less lonely than individuals without these feelings. Consequently, individuals may transfer social relationships to the virtual world, treating them as more secure than social contacts in the real world. In this context, the mistrust/abuse schema may promote a vulnerability to external stress associated with social relationships. In turn, high levels of external stress may contribute to PFU development. Previous studies’ findings indicate a positive relationship between social anxiety and PFU [19,92] and seems to support this assumption. The first mechanism may be related to a lack of caution in online interactions with others when an individual engages in a dependency relationship between them and others on the Facebook platform. This assumption supports other results in the present study, indicating a positive relationship between PFU and (i) dependence/incompetence schema and (ii) enmeshment schema. More specifically, in order to satisfy emotional needs, an individual may enter into entangled relationships with other Facebook users, believing that they present no threat to them. However, these assumptions require further research.

### 4.6. Other Indirect Effects between the EMSs and PFU via Perceived Stress

The findings also showed a positive indirect effect between the failure to achieve schema and PFU via external stress. More specifically, individuals who believe it is impossible to achieve as much as others due to poorer competence/ability may present higher external stress, particularly in social situations. Higher external stress levels may contribute to a greater propensity for PFU. In this context, previous research [88] has found a negative relationship between social competence and PFU. Moreover, Pontes et al. [94] reported that social networking site addiction was positively associated with the belief that an individual is safer, more effective, more confident, and more comfortable in interpersonal interactions online than in traditional face-to-face social activities. Additionally, Pontes et al. [94] reported a positive relationship between maladaptive cognitions, fear of missing out, dysfunctional emotion regulation, general psychiatric distress, and social networking site addiction. Consequently, it can be assumed that the belief that individuals lack social competence may be linked to higher stress levels in external situations involving social relationships. Continuing higher stress levels may contribute to choosing online rather than offline contacts as more secure, consequently increasing engagement in Facebook use.

There was a negative indirect effect between self-punitiveness and PFU via external stress. More specifically, individuals who believe every mistake deserves severe punishment present less vulnerability to external stress. Consequently, it may be assumed that this schema may indirectly contribute to reducing stress, and therefore decrease preoccupation with Facebook. Additionally, it should be noted that self-punitiveness was negatively associated with all perceived stress dimensions. The self-punitiveness schema is a part of the excessive responsibility and standards dimension. This dimension concerns the parent–child relationship, which is dominated by high expectations of the child, often impossible to meet, and a perfectionist approach to achievements (see [23,28]). In this parent–child relationship, the relationship is dominated by punishment for offences, an undervaluing of successes, and a heightened responsibility (see [23,28]). Consequently, it can be assumed that individuals with a self-punitiveness schema may avoid stressful situations or try to control them so that they do not experience any negative effects. More specifically, when an individual believes that every mistake deserves severe punishment, they may avoid a stressful situation in order not to experience negative effects related to the possibility of making a mistake, or they may try to control the stressful situation in order not to make a mistake. However, further research is needed to verify this speculation.

### 4.7. The Relationship between EMSs and Perceived Stress

**Figure 1:**
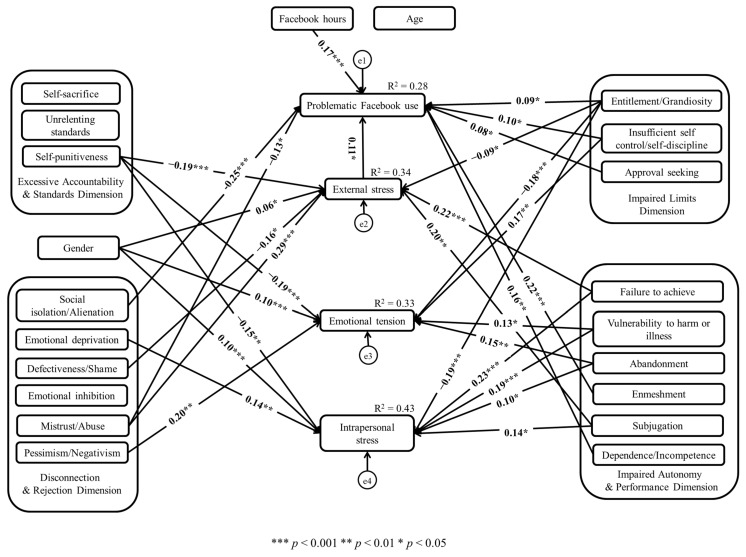
Statistically significant paths between analyzed variables.

Moreover, different patterns of relationships between EMSs and perceived stress dimensions were observed (see Figure 1). The findings were partly consistent with previous research [37], indicating a positive relationship between social stressors and EMSs included in the disconnection/rejection schema dimension. Additionally, the present study’s results also showed that perceived stress dimensions were associated with schemas from impaired limits and impaired autonomy and performance dimensions. There was also a negative relationship between all perceived stress dimensions and the self-punitiveness schema, which is a part of the excessive responsibility and standards schema dimension. Consequently, it can be assumed that different pattern configurations may be associated with various dimensions of stress. However, more rigorous research is needed to understand these relationships more broadly.

## 5. Limitations

The results of the study should be interpreted taking into account some limitations. First, the study was cross-sectional, so causal relationships cannot be inferred from the data presented. Second, the participants completed self-report methods, so various methods biases may have occurred. More specifically, in surveys, participants can give socially acceptable answers, avoid answering difficult questions, and/or not focus enough attention on question content. Cheng et al. [6] identified cross-cultural differences in social media addiction prevalence. For example, collectivist nations have higher social media addiction prevalence rates than individualist nations. Consequently, caution should be exercised in generalizing the results presented in the present study to different cultures. Moreover, participants recruited online from the Polish research panel *Ariadna* may have responded in a biased manner. Additionally, the participants’ ages ranged from 18 to 35 years. Consequently, caution should be exercised when generalizing the results obtained to other age groups, such as children or seniors.

## 6. Conclusions

The present study’s findings confirmed previous research [49] indicating that PFU was mostly positively associated with EMSs included in the impaired limits domain. Additionally, there was also a positive relationship between the PFU and EMSs included in the impaired autonomy and performance domain (dependence/incompetence and enmeshment schemas). The findings showed that perceived external stress mediated the relationship between EMSs and PFU. These findings may be important for understanding the mechanisms underlying PFU development related to the response to stressful situations. Additionally, knowing the EMSs associated with PFU and perceived stress might improve the therapeutic interventions and prevention of this problematic behavior.

More specifically, schema therapy [21] is one of the methods used in behavioral addiction treatment. Schema therapy [23,26] is an integrative approach based on cognitive behavioral therapy (CBT), attachment, object relations, Gestalt theories, and other psychotherapeutic approaches. This therapy aims to break maladaptive patterns of thinking, feeling, and behaving and to develop healthier alternatives using therapeutic techniques from CBT, Gestalt, and other psychotherapeutic approaches. More specifically, during this therapy, individuals recognize and understand the causes of their behaviors. Additionally, individuals change their thoughts and behaviors to better cope with social relationships or emotional challenges in a healthy way. Consequently, understanding the exact relationship between EMSs and PFU may enable the development of more effective therapies and prevention methods based on Young et al.’s [23] model.

## Figures and Tables

**Table 2:** Sample characteristics (N = 993).

Variable	Category	Sample (N = 933)	
		N	Percentage
Gender	Female	505	50.86
	Male	488	49.14
Education	Primary education	16	1.61
	Vocational education	52	5.24
	Secondary education	314	31.62
	Post-secondary education	113	11.38
	University education	498	53.38
Residence	Village	242	24.37
	Small city (up to 20,000 residents)	123	12.39
	Medium city (between 20,000 and 100,000 residents)	219	22.05
	Large city (above 100,000 residents)	409	41.19
Marital status	Single	287	28.90
	In a relationship	330	33.23
	In a married relationship	361	36.35
	Widowed	1	0.10
	Divorced	14	1.41

**Table 3:** The relationship between problematic Facebook use, perceived stress dimensions, and other analyzed variables.

Variables	Category	Descriptive Statistics		Problematic Facebook Use			Perceived Stress Dimensions								
							Intrapsychic Stress			External Stress			Emotional Tension		
		M	SD	rho	95% CI		rho	95% CI		rho	95% CI		rho	95% CI	
					Lower	Upper		Lower	Upper		Lower	Upper		Lower	Upper
Age		27.38	4.79	−0.01	−0.07	0.06	−0.12 ***	−0.18	−0.05	−0.10 **	−0.16	−0.03	−0.10 **	−0.16	−0.04
Gender (0: male; 1: female)		0.51	0.50	0.02	−0.05	0.08	0.15 ***	0.08	0.21	0.11 **	0.05	0.17	0.17 ***	0.10	0.23
Hours spent using Facebook		14.33	19.68	0.33 ***	0.28	0.39	0.08 *	0.01	0.14	0.10 **	0.04	0.16	0.09 **	0.03	0.16
Early maladaptive schemas	Emotional deprivation	2.61	1.18	0.25 ***	0.18	0.31	0.49 ***	0.44	0.54	0.43 ***	0.38	0.48	0.33 ***	0.27	0.39
	Abandonment	2.97	1.03	0.27 ***	0.21	0.33	0.47 ***	0.42	0.52	0.40 ***	0.35	0.46	0.43 ***	0.38	0.48
	Mistrust/abuse	2.86	1.03	0.22 ***	0.16	0.28	0.50 ***	0.45	0.55	0.49 ***	0.44	0.54	0.38 ***	0.33	0.44
	Social isolation/alienation	2.97	1.11	0.11 ***	0.05	0.18	0.48 ***	0.43	0.53	0.42 ***	0.36	0.47	0.38 ***	0.32	0.43
	Defectiveness/shame	2.41	1.20	0.26 ***	0.20	0.32	0.48 ***	0.43	0.53	0.41 ***	0.35	0.46	0.34 ***	0.28	0.40
	Failure to achieve	2.77	1.10	0.28 ***	0.22	0.33	0.56 ***	0.51	0.60	0.47 ***	0.42	0.52	0.41 ***	0.36	0.46
	Dependence/incompetence	2.55	0.98	0.35 ***	0.29	0.40	0.49 ***	0.44	0.54	0.44 ***	0.39	0.49	0.40 ***	0.34	0.45
	Vulnerability to harm or illness	2.80	1.03	0.30 ***	0.24	0.35	0.51 ***	0.47	0.56	0.45 ***	0.40	0.50	0.42 ***	0.37	0.47
	Enmeshment	2.39	1.06	0.37 ***	0.32	0.43	0.35 ***	0.30	0.41	0.35 ***	0.29	0.40	0.24 ***	0.18	0.30
	Subjugation	2.68	1.01	0.29 ***	0.23	0.35	0.52 ***	0.48	0.57	0.48 ***	0.43	0.53	0.40 ***	0.34	0.45
	Self-sacrifice	3.21	0.85	0.18 ***	0.12	0.24	0.25 ***	0.19	0.31	0.25 ***	0.19	0.31	0.22 ***	0.16	0.28
	Emotional inhibition	2.86	1.07	0.18 ***	0.12	0.24	0.44 ***	0.38	0.49	0.38 ***	0.33	0.43	0.35 ***	0.29	0.40
	Unrelenting standards	3.13	0.88	0.21 ***	0.14	0.27	0.31 ***	0.25	0.37	0.31 ***	0.25	0.37	0.28 ***	0.22	0.34
	Entitlement/grandiosity	2.90	0.85	0.27 ***	0.21	0.33	0.21 ***	0.15	0.27	0.28 ***	0.22	0.33	0.19 ***	0.13	0.25
	Insufficient self-control/self-discipline	2.86	0.95	0.30 ***	0.24	0.36	0.45 ***	0.40	0.50	0.39 ***	0.34	0.45	0.40 ***	0.34	0.45
	Approval seeking	3.10	0.96	0.30 ***	0.24	0.36	0.32 ***	0.26	0.38	0.32 ***	0.26	0.38	0.31 ***	0.25	0.37
	Pessimism/negativism	3.04	1.05	0.22 ***	0.16	0.28	0.52 ***	0.47	0.57	0.47 ***	0.42	0.52	0.46 ***	0.41	0.51
	Self-punitiveness	2.62	0.99	0.25 ***	0.19	0.31	0.49 ***	0.44	0.54	0.32 ***	0.26	0.37	0.26 ***	0.20	0.32
PS	Emotional tension	3.07	0.86	0.23 ***	0.16	0.29	0.73 ***	0.69	0.76	0.66 ***	0.62	0.70			
	External stress	2.89	0.72	0.27 ***	0.21	0.33	0.71 ***	0.67	0.74						
	Intrapsychic stress	2.96	0.80	0.25 ***	0.19	0.31									

Note: *** *p* < 0.001, ** *p* < 0.01, * *p* < 0.05; PS = perceived stress; CI = confidence interval.

**Table 4:** Standardized path coefficients for perceived stress dimensions and problematic Facebook use among Facebook users (N = 993).

Variables	Category	Perceived Stress									To Problematic Facebook Use		
		To Emotional Tension			To External Stress			To Intrapsychic Stress					
		β	SE	*p*	β	SE	*p*	β	SE	*p*	β	SE	*p*
Early maladaptive schemas	Emotional deprivation	0.05	0.06	0.335	0.09	0.05	0.076	**0.14**	**0.05**	**0.005**	0.07	0.05	0.227
	Abandonment	**0.15**	**0.05**	**0.006**	0.01	0.05	0.960	**0.10**	**0.05**	**0.027**	0.01	0.05	0.986
	Mistrust/abuse	0.01	0.07	0.962	**0.29**	**0.06**	**0.001**	0.09	0.06	0.133	**−0.13**	**0.06**	**0.040**
	Social isolation/alienation	0.06	0.05	0.207	0.03	0.05	0.552	0.07	0.05	0.108	**−0.25**	**0.05**	**0.001**
	Defectiveness/shame	−0.05	0.06	0.472	**−0.16**	**0.06**	**0.010**	−0.04	0.05	0.466	−0.09	0.06	0.175
	Failure to achieve	0.05	0.06	0.439	**0.22**	**0.05**	**0.001**	**0.23**	**0.05**	**0.001**	0.01	0.05	0.873
	Dependence/incompetence	0.11	0.06	0.082	0.06	0.06	0.328	0.00	0.05	0.957	**0.16**	**0.06**	**0.009**
	Vulnerability to harm or illness	**0.13**	**0.06**	**0.039**	0.08	0.06	0.179	**0.19**	**0.05**	**0.001**	0.07	0.06	0.219
	Enmeshment	−0.08	0.05	0.108	0.03	0.05	0.519	−0.03	0.04	0.415	**0.22**	**0.04**	**0.001**
	Subjugation	0.07	0.07	0.282	**0.20**	**0.06**	**0.001**	**0.14**	**0.06**	**0.012**	0.01	0.07	0.986
	Self-sacrifice	−0.03	0.04	0.443	−0.07	0.04	0.081	−0.05	0.04	0.131	0.03	0.04	0.463
	Emotional inhibition	−0.03	0.05	0.531	−0.08	0.05	0.099	−0.04	0.04	0.417	−0.04	0.05	0.428
	Unrelenting standards	0.04	0.05	0.420	0.03	0.05	0.536	0.02	0.04	0.636	0.02	0.05	0.681
	Entitlement/grandiosity	**−0.18**	**0.04**	**0.001**	**−0.09**	**0.04**	**0.034**	**−0.19**	**0.04**	**0.001**	**0.09**	**0.04**	**0.018**
	Insufficient self-control/self-discipline	**0.17**	**0.05**	**0.001**	0.01	0.05	0.774	0.08	0.04	0.084	**0.10**	**0.05**	**0.044**
	Approval seeking	0.02	0.05	0.644	0.01	0.04	0.985	−0.02	0.04	0.647	**0.08**	**0.04**	**0.039**
	Pessimism/negativism	**0.20**	**0.06**	**0.002**	0.07	0.06	0.207	0.07	0.06	0.234	−0.11	0.06	0.070
	Self-punitiveness	**−0.19**	**0.05**	**0.001**	**−0.19**	**0.05**	**0.001**	**−0.15**	**0.05**	**0.002**	0.03	0.05	0.507
Age		−0.02	0.03	0.425	−0.03	0.03	0.214	−0.02	0.03	0.335	0.05	0.03	0.078
Gender (0: male; 1: female)		**0.10**	**0.03**	**0.001**	**0.06**	**0.03**	**0.034**	**0.10**	**0.03**	**0.001**	−0.02	0.03	0.597
PS	Emotional tension										0.09	0.05	0.073
	External stress										**0.11**	**0.05**	**0.016**
	Intrapsychic stress										0.06	0.05	0.239
Hours spent using Facebook											**0.17**	**0.03**	**0.001**

Note: PS = perceived stress; emboldened results = statistically significant.

**Table 5:** Standardized indirect effects exerted by external stress in relationship between EMSs and PFU with 95% confidence intervals (CIs) among Facebook users (N = 993).

Pathways	Point Estimates	Standard Error	z	*p*	95% CIs	
					Lower	Upper
Emotional deprivation → External stress → PFU	0.010	0.008	1.33	0.183	−0.001	0.028
Abandonment → External stress → PFU	−0.001	0.006	−0.06	0.950	−0.013	0.012
**Mistrust/abuse → External stress → PFU**	**0.032**	**0.015**	**2.08**	**0.038**	**0.005**	**0.065**
Social isolation/alienation → External stress → PFU	0.003	0.006	0.52	0.606	−0.008	0.017
Defectiveness/shame → External stress → PFU	−0.018	0.011	−1.70	0.089	−0.042	−0.001
**Failure to achieve → External stress → PFU**	**0.024**	**0.012**	**2.04**	**0.042**	**0.003**	**0.050**
Dependence/incompetence → External stress → PFU	0.006	0.008	0.82	0.410	−0.007	0.024
Vulnerability to harm or illness → External stress → PFU	0.009	0.008	1.08	0.280	−0.004	0.028
Enmeshment → External stress → PFU	0.003	0.006	0.56	0.577	−0.007	0.017
Subjugation → External stress → PFU	0.022	0.012	1.84	0.066	0.003	0.050
Self-sacrifice → External stress → PFU	−0.007	0.005	−1.35	0.178	−0.020	0.001
Emotional inhibition → External stress → PFU	−0.009	0.007	−1.30	0.195	−0.025	0.002
Unrelenting standards → External stress → PFU	0.003	0.006	0.54	0.592	−0.008	0.016
Entitlement/grandiosity → External stress → PFU	−0.010	0.006	−1.53	0.127	−0.025	0.001
Insufficient self-control/self-discipline → External stress → PFU	0.001	0.006	0.25	0.806	−0.010	0.014
Approval seeking → External stress → PFU	−0.001	0.005	−0.03	0.973	−0.011	0.010
Pessimism/negativism → External stress → PFU	0.008	0.008	1.03	0.305	−0.005	0.026
**Self-punitiveness → External stress → PFU**	**−0.021**	**0.011**	**−1.98**	**0.048**	**−0.045**	**−0.003**

Note: PFU = problematic Facebook use; emboldened variables = statistically significant.

## Data Availability

The dataset from the present study is available from the John Paul II Catholic University of Lublin repository database (access link: http://hdl.handle.net/20.500.12153/3890).

## Supplementary Materials

The following supporting information can be downloaded at: https://www.mdpi.com/article/10.3390/ijerph20042969/s1, Table S1: The items of Facebook Intrusion Questionnaire; Table S2: Correlations between early maladaptive schemas, age, gender, and hours spent using Facebook, and between residuals of perceived stress dimensions; Table S3: Standardized indirect effects exerted by emotional tension and intrapersonal stress in relationship between EMSs and PFU with 95% confidence intervals (CIs) among Facebook users (N = 993).
