# Supplementary material for: The Relationship between Early Maladaptive Schemas and Problematic Facebook Use: The Indirect Effects of Perceived Stress
Source: Int J Environ Res Public Health. 2023 Feb 8;20(4):2969. doi: 10.3390/ijerph20042969 (PMC9957475; doi:10.3390/ijerph20042969)
Supplement: Supplementary file 1 [file ijerph-20-02969-s001.zip › ijerph-2073240-supplementary/Table S1.pdf]

Table S1. The items of Facebook Intrusion Questionnaire.

Items
1. I often think about Facebook when I am not using it.
2. I often use Facebook for no particular reason.
3. Arguments have arisen with others because of my Facebook use.
4. I interrupt whatever else I am doing when I feel the need to access Facebook.
5. I feel connected to others when I use Facebook.
6. I lose track of how much I am using Facebook.
7. The thought of not being able to access Facebook makes me feel distressed.
8. I have been unable to reduce my Facebook use.
