# Supplementary material for: The Relationship between Early Maladaptive Schemas and Problematic Facebook Use: The Indirect Effects of Perceived Stress
Source: Int J Environ Res Public Health. 2023 Feb 8;20(4):2969. doi: 10.3390/ijerph20042969 (PMC9957475; doi:10.3390/ijerph20042969)
Supplement: Supplementary file 1 [file ijerph-20-02969-s001.zip › ijerph-2073240-supplementary/Table S2.pdf]

Table S2. Correlations between early maladaptive schemas, age, gender and hour sent using Facebook, and between residuals of perceived stress dimensions.

Pathways	r	SE	z	*p*	95% CI	
					Lower	Upper.
Intrapsychic stress - External stress	0.61	0.02	26.86	0.001	0.57	0.66
Intrapsychic stress - Emotional tension	0.66	0.02	30.76	0.001	0.62	0.70
External stress - Emotional tension	0.60	0.02	24.99	0.001	0.56	0.65
Emotional deprivation - Abandonment	0.59	0.03	22.07	0.001	0.54	0.65
Emotional deprivation - Mistrust/Abuse	0.77	0.02	50.90	0.001	0.74	0.80
Emotional deprivation - Social isolation/Alienation	0.70	0.02	33.14	0.001	0.66	0.74
Emotional deprivation - Defectiveness/Shame	0.80	0.02	51.56	0.001	0.77	0.83
Emotional deprivation - Failure to achieve	0.70	0.02	35.19	0.001	0.66	0.74
Emotional deprivation - Dependence/Incompetence	0.71	0.02	35.31	0.001	0.67	0.75
Emotional deprivation - Vulnerability to harm or illness	0.65	0.02	27.17	0.001	0.60	0.70
Emotional deprivation - Enmeshment	0.65	0.02	26.39	0.001	0.60	0.69
Emotional deprivation - Subjugation	0.77	0.02	47.07	0.001	0.74	0.80
Emotional deprivation - Self-sacrifice	0.45	0.03	14.37	0.001	0.39	0.51
Emotional deprivation - Emotional inhibition	0.66	0.02	30.21	0.001	0.62	0.71
Emotional deprivation - Unrelenting standards	0.50	0.03	17.34	0.001	0.44	0.55
Emotional deprivation - Entitlement/Grandiosity	0.49	0.03	16.66	0.001	0.44	0.55
Emotional deprivation - Insufficient self-control/self-discipline	0.60	0.02	23.95	0.001	0.55	0.65
Emotional deprivation - Approval seeking	0.47	0.03	15.79	0.001	0.42	0.53
Emotional deprivation - Pessimism/Negativism	0.65	0.02	29.47	0.001	0.61	0.70
Emotional deprivation - Self-punitiveness	0.66	0.02	28.37	0.001	0.61	0.70
Emotional deprivation - Age	-0.03	0.03	-1.03	0.305	-0.09	0.03
Emotional deprivation - Hours spent using Facebook	0.05	0.03	1.61	0.108	-0.01	0.10
Emotional deprivation - Gender	-0.06	0.03	-1.85	0.064	-0.12	0.00
Abandonment - Mistrust/Abuse	0.72	0.02	34.05	0.001	0.68	0.76
Abandonment - Social isolation/Alienation	0.59	0.03	21.58	0.001	0.54	0.65
Abandonment - Defectiveness/Shame	0.66	0.02	28.37	0.001	0.61	0.70
Abandonment - Failure to achieve	0.67	0.02	28.11	0.001	0.63	0.72
Abandonment - Dependence/Incompetence	0.70	0.02	33.74	0.001	0.66	0.74
Abandonment - Vulnerability to harm or illness	0.77	0.02	44.65	0.001	0.73	0.80
Abandonment - Enmeshment	0.58	0.03	21.90	0.001	0.53	0.64
Abandonment - Subjugation	0.73	0.02	40.20	0.001	0.70	0.77
Abandonment - Self-sacrifice	0.53	0.03	18.19	0.001	0.47	0.59
Abandonment - Emotional inhibition	0.58	0.03	20.51	0.001	0.52	0.63
Abandonment - Unrelenting standards	0.61	0.03	24.14	0.001	0.56	0.66
Abandonment - Entitlement/Grandiosity	0.54	0.03	18.67	0.001	0.48	0.59
Abandonment - Insufficient self-control/self-discipline	0.63	0.03	24.63	0.001	0.58	0.68
Abandonment - Approval seeking	0.63	0.02	25.47	0.001	0.58	0.68
Abandonment - Pessimism/Negativism	0.75	0.02	38.15	0.001	0.71	0.78
Abandonment - Self-punitiveness	0.64	0.02	25.48	0.001	0.59	0.68
Abandonment - Age	-0.12	0.03	-3.68	0.001	-0.18	-0.05
Abandonment - Hours spent using Facebook	0.06	0.03	1.69	0.091	-0.01	0.12

Abandonment - Gender	0.07	0.03	2.09	0.036	0.00	0.13
Mistrust/Abuse - Social isolation/Alienation	0.74	0.02	41.95	0.001	0.71	0.78
Mistrust/Abuse - Defectiveness/Shame	0.75	0.02	42.87	0.001	0.72	0.79
Mistrust/Abuse - Failure to achieve	0.71	0.02	34.31	0.001	0.67	0.75
Mistrust/Abuse - Dependence/Incompetence	0.71	0.02	33.04	0.001	0.67	0.75
Mistrust/Abuse - Vulnerability to harm or illness	0.78	0.02	50.90	0.001	0.75	0.81
Mistrust/Abuse - Enmeshment	0.62	0.02	24.81	0.001	0.57	0.67
Mistrust/Abuse - Subjugation	0.79	0.01	54.45	0.001	0.76	0.81
Mistrust/Abuse - Self-sacrifice	0.55	0.03	20.23	0.001	0.50	0.61
Mistrust/Abuse - Emotional inhibition	0.69	0.02	31.95	0.001	0.65	0.73
Mistrust/Abuse - Unrelenting standards	0.65	0.02	26.57	0.001	0.61	0.70
Mistrust/Abuse - Entitlement/Grandiosity	0.59	0.03	21.25	0.001	0.54	0.65
Mistrust/Abuse - Insufficient self-control/self-discipline	0.66	0.02	27.98	0.001	0.62	0.71
Mistrust/Abuse - Approval seeking	0.60	0.03	21.34	0.001	0.54	0.65
Mistrust/Abuse - Pessimism/Negativism	0.82	0.01	61.25	0.001	0.79	0.84
Mistrust/Abuse - Self-punitiveness	0.71	0.02	35.77	0.001	0.67	0.75
Mistrust/Abuse – Age	-0.06	0.03	-2.05	0.040	-0.13	0.00
Mistrust/Abuse - Hours spent using Facebook	0.02	0.03	0.56	0.579	-0.05	0.08
Mistrust/Abuse – Gender	-0.01	0.03	-0.29	0.774	-0.07	0.05
Social isolation/Alienation - Defectiveness/Shame	0.66	0.02	28.92	0.001	0.61	0.70
Social isolation/Alienation - Failure to achieve	0.70	0.02	30.78	0.001	0.66	0.75
Social isolation/Alienation - Dependence/Incompetence	0.65	0.02	28.30	0.001	0.61	0.70
Social isolation/Alienation - Vulnerability to harm or illness	0.61	0.02	24.77	0.001	0.57	0.66
Social isolation/Alienation – Enmeshment	0.53	0.03	19.41	0.001	0.48	0.59
Social isolation/Alienation – Subjugation	0.70	0.02	35.50	0.001	0.67	0.74
Social isolation/Alienation - Self-sacrifice	0.40	0.03	11.89	0.001	0.34	0.47
Social isolation/Alienation - Emotional inhibition	0.76	0.02	44.26	0.001	0.72	0.79
Social isolation/Alienation - Unrelenting standards	0.52	0.03	17.82	0.001	0.47	0.58
Social isolation/Alienation - Entitlement/Grandiosity	0.49	0.03	15.99	0.001	0.43	0.55
Social isolation/Alienation - Insufficient self-control/self-discipline	0.63	0.02	26.38	0.001	0.59	0.68
Social isolation/Alienation - Approval seeking	0.44	0.03	13.27	0.001	0.38	0.51
Social isolation/Alienation - Pessimism/Negativism	0.69	0.02	29.99	0.001	0.64	0.73
Social isolation/Alienation - Self-punitiveness	0.62	0.02	25.71	0.001	0.57	0.66
Social isolation/Alienation – Age	-0.08	0.03	-2.75	0.006	-0.14	-0.02
Social isolation/Alienation - Hours spent using Facebook	-0.03	0.03	-0.98	0.325	-0.09	0.03
Social isolation/Alienation – Gender	0.01	0.03	0.20	0.841	-0.06	0.07
Defectiveness/Shame - Failure to achieve	0.76	0.02	45.33	0.001	0.73	0.79
Defectiveness/Shame - Dependence/Incompetence	0.79	0.02	49.06	0.001	0.76	0.82
Defectiveness/Shame - Vulnerability to harm or illness	0.69	0.02	31.98	0.001	0.65	0.73
Defectiveness/Shame – Enmeshment	0.70	0.02	32.23	0.001	0.66	0.75
Defectiveness/Shame – Subjugation	0.80	0.02	52.06	0.001	0.77	0.83
Defectiveness/Shame - Self-sacrifice	0.36	0.03	10.48	0.001	0.29	0.43
Defectiveness/Shame - Emotional inhibition	0.69	0.02	34.01	0.001	0.65	0.73
Defectiveness/Shame - Unrelenting standards	0.49	0.03	16.24	0.001	0.43	0.55
Defectiveness/Shame - Entitlement/Grandiosity	0.45	0.03	13.60	0.001	0.38	0.51

Defectiveness/Shame - Insufficient self-control/self-discipline	0.65	0.02	28.83	0.001	0.61	0.70
Defectiveness/Shame - Approval seeking	0.49	0.03	16.12	0.001	0.43	0.55
Defectiveness/Shame - Pessimism/Negativism	0.67	0.02	28.76	0.001	0.62	0.71
Defectiveness/Shame - Self-punitiveness	0.77	0.02	50.52	0.001	0.74	0.80
Defectiveness/Shame – Age	-0.11	0.03	-3.64	0.001	-0.17	-0.05
Defectiveness/Shame - Hours spent using Facebook	0.05	0.03	1.61	0.107	-0.01	0.11
Defectiveness/Shame – Gender	-0.07	0.03	-2.22	0.026	-0.13	-0.01
Failure to achieve - Dependence/Incompetence	0.79	0.02	52.52	0.001	0.76	0.82
Failure to achieve - Vulnerability to harm or illness	0.73	0.02	35.72	0.001	0.69	0.77
Failure to achieve – Enmeshment	0.61	0.03	23.18	0.001	0.56	0.66
Failure to achieve – Subjugation	0.78	0.02	44.25	0.001	0.75	0.81
Failure to achieve - Self-sacrifice	0.41	0.03	12.13	0.001	0.35	0.48
Failure to achieve - Emotional inhibition	0.69	0.02	29.35	0.001	0.64	0.73
Failure to achieve - Unrelenting standards	0.51	0.03	16.34	0.001	0.45	0.57
Failure to achieve - Entitlement/Grandiosity	0.45	0.03	13.63	0.001	0.39	0.52
Failure to achieve - Insufficient self-control/self-discipline	0.74	0.02	42.57	0.001	0.71	0.77
Failure to achieve - Approval seeking	0.54	0.03	18.44	0.001	0.48	0.59
Failure to achieve - Pessimism/Negativism	0.74	0.02	36.99	0.001	0.70	0.78
Failure to achieve - Self-punitiveness	0.73	0.02	35.73	0.001	0.69	0.77
Failure to achieve – Age	-0.13	0.03	-4.12	0.001	-0.19	-0.07
Failure to achieve - Hours spent using Facebook	0.06	0.03	1.76	0.078	-0.01	0.12
Failure to achieve – Gender	0.05	0.03	1.48	0.139	-0.02	0.11
Dependence/Incompetence - Vulnerability to harm or illness	0.74	0.02	40.85	0.001	0.70	0.77
Dependence/Incompetence – Enmeshment	0.75	0.02	39.59	0.001	0.71	0.78
Dependence/Incompetence – Subjugation	0.82	0.01	56.43	0.001	0.79	0.85
Dependence/Incompetence - Self-sacrifice	0.41	0.04	11.21	0.001	0.33	0.48
Dependence/Incompetence - Emotional inhibition	0.69	0.02	35.28	0.001	0.65	0.73
Dependence/Incompetence - Unrelenting standards	0.50	0.03	16.21	0.001	0.44	0.57
Dependence/Incompetence - Entitlement/Grandiosity	0.52	0.03	17.59	0.001	0.46	0.58
Dependence/Incompetence - Insufficient self-control/self-discipline	0.74	0.02	39.71	0.001	0.70	0.78
Dependence/Incompetence - Approval seeking	0.56	0.03	20.75	0.001	0.51	0.62
Dependence/Incompetence - Pessimism/Negativism	0.69	0.02	33.47	0.001	0.65	0.73
Dependence/Incompetence - Self-punitiveness	0.72	0.02	34.31	0.001	0.68	0.76
Dependence/Incompetence – Age	-0.12	0.03	-4.00	0.001	-0.19	-0.06
Dependence/Incompetence - Hours spent using Facebook	0.08	0.03	2.42	0.015	0.02	0.15
Dependence/Incompetence – Gender	0.01	0.03	0.28	0.776	-0.05	0.07
Vulnerability to harm or illness - Enmeshment	0.64	0.02	25.85	0.001	0.59	0.69
Vulnerability to harm or illness - Subjugation	0.75	0.02	42.09	0.001	0.71	0.78
Vulnerability to harm or illness - Self-sacrifice	0.50	0.03	16.88	0.001	0.45	0.56
Vulnerability to harm or illness - Emotional inhibition	0.62	0.03	24.86	0.001	0.57	0.67
Vulnerability to harm or illness - Unrelenting standards	0.61	0.03	23.63	0.001	0.56	0.66
Vulnerability to harm or illness - Entitlement/Grandiosity	0.56	0.03	20.03	0.001	0.51	0.62
Vulnerability to harm or illness - Insufficient self-control/self-discipline	0.66	0.02	29.81	0.001	0.62	0.71
Vulnerability to harm or illness - Approval seeking	0.58	0.03	21.55	0.001	0.53	0.63

Vulnerability to harm or illness - Pessimism/Negativism	0.84	0.01	75.34	0.001	0.82	0.86
Vulnerability to harm or illness - Self-punitiveness	0.68	0.02	29.66	0.001	0.63	0.72
Vulnerability to harm or illness - Age	-0.06	0.03	-1.75	0.081	-0.12	0.01
Vulnerability to harm or illness - Hours spent using Facebook	0.07	0.03	2.17	0.030	0.01	0.14
Vulnerability to harm or illness - Gender	0.04	0.03	1.40	0.162	-0.02	0.11
Enmeshment – Subjugation	0.70	0.02	33.20	0.001	0.66	0.74
Enmeshment - Self-sacrifice	0.42	0.03	12.92	0.001	0.36	0.49
Enmeshment - Emotional inhibition	0.57	0.03	22.25	0.001	0.52	0.62
Enmeshment - Unrelenting standards	0.49	0.03	16.34	0.001	0.43	0.55
Enmeshment - Entitlement/Grandiosity	0.55	0.03	18.94	0.001	0.49	0.61
Enmeshment - Insufficient self-control/self-discipline	0.59	0.03	20.73	0.001	0.53	0.64
Enmeshment - Approval seeking	0.48	0.03	16.15	0.001	0.42	0.54
Enmeshment - Pessimism/Negativism	0.54	0.03	18.46	0.001	0.48	0.59
Enmeshment - Self-punitiveness	0.66	0.02	27.63	0.001	0.61	0.71
Enmeshment – Age	-0.06	0.03	-1.92	0.055	-0.12	0.00
Enmeshment - Hours spent using Facebook	0.12	0.04	3.33	0.001	0.05	0.19
Enmeshment – Gender	-0.05	0.03	-1.56	0.118	-0.11	0.01
Subjugation - Self-sacrifice	0.50	0.03	16.39	0.001	0.44	0.56
Subjugation - Emotional inhibition	0.73	0.02	39.18	0.001	0.70	0.77
Subjugation - Unrelenting standards	0.62	0.03	24.63	0.001	0.57	0.67
Subjugation - Entitlement/Grandiosity	0.55	0.03	18.72	0.001	0.49	0.60
Subjugation - Insufficient self-control/self-discipline	0.71	0.02	32.79	0.001	0.67	0.75
Subjugation - Approval seeking	0.60	0.03	22.23	0.001	0.54	0.65
Subjugation - Pessimism/Negativism	0.76	0.02	47.63	0.001	0.73	0.79
Subjugation - Self-punitiveness	0.74	0.02	38.99	0.001	0.71	0.78
Subjugation – Age	-0.09	0.03	-2.72	0.006	-0.15	-0.02
Subjugation - Hours spent using Facebook	0.02	0.03	0.53	0.597	-0.04	0.08
Subjugation – Gender	0.00	0.03	-0.07	0.945	-0.06	0.06
Self-sacrifice - Emotional inhibition	0.31	0.04	8.09	0.001	0.23	0.38
Self-sacrifice - Unrelenting standards	0.59	0.03	20.66	0.001	0.53	0.64
Self-sacrifice - Entitlement/Grandiosity	0.49	0.03	14.81	0.001	0.43	0.56
Self-sacrifice - Insufficient self-control/self-discipline	0.39	0.04	10.90	0.001	0.32	0.46
Self-sacrifice - Approval seeking	0.45	0.03	13.22	0.001	0.38	0.52
Self-sacrifice - Pessimism/Negativism	0.52	0.03	17.27	0.001	0.46	0.58
Self-sacrifice - Self-punitiveness	0.46	0.03	13.59	0.001	0.39	0.53
Self-sacrifice – Age	0.04	0.03	1.12	0.264	-0.03	0.10
Self-sacrifice - Hours spent using Facebook	0.05	0.03	1.78	0.074	-0.01	0.11
Self-sacrifice – Gender	0.11	0.03	3.60	0.001	0.05	0.17
Emotional inhibition - Unrelenting standards	0.52	0.03	17.22	0.001	0.46	0.57
Emotional inhibition - Entitlement/Grandiosity	0.47	0.03	14.17	0.001	0.40	0.53
Emotional inhibition - Insufficient self-control/self-discipline	0.65	0.02	26.87	0.001	0.61	0.70
Emotional inhibition - Approval seeking	0.46	0.03	14.53	0.001	0.40	0.52
Emotional inhibition - Pessimism/Negativism	0.67	0.02	29.54	0.001	0.63	0.72
Emotional inhibition - Self-punitiveness	0.62	0.03	23.82	0.001	0.56	0.67
Emotional inhibition - Age	-0.07	0.03	-2.32	0.020	-0.13	-0.01

Emotional inhibition - Hours spent using Facebook	0.00	0.04	0.03	0.974	-0.07	0.07
Emotional inhibition - Gender	-0.03	0.03	-0.97	0.332	-0.09	0.03
Unrelenting standards - Entitlement/Grandiosity	0.59	0.03	21.66	0.001	0.54	0.65
Unrelenting standards - Insufficient self-control/self-discipline	0.50	0.03	14.56	0.001	0.44	0.57
Unrelenting standards - Approval seeking	0.65	0.02	26.37	0.001	0.60	0.70
Unrelenting standards - Pessimism/Negativism	0.65	0.03	24.74	0.001	0.60	0.70
Unrelenting standards - Self-punitiveness	0.65	0.02	27.32	0.001	0.60	0.70
Unrelenting standards - Age	-0.05	0.03	-1.51	0.131	-0.11	0.01
Unrelenting standards - Hours spent using Facebook	-0.03	0.03	-0.83	0.405	-0.09	0.04
Unrelenting standards - Gender	-0.01	0.03	-0.29	0.768	-0.07	0.05
Entitlement/Grandiosity - Insufficient self-control/self-discipline	0.56	0.03	19.55	0.001	0.51	0.62
Entitlement/Grandiosity - Approval seeking	0.64	0.02	26.71	0.001	0.59	0.68
Entitlement/Grandiosity - Pessimism/Negativism	0.51	0.03	17.21	0.001	0.46	0.57
Entitlement/Grandiosity - Self-punitiveness	0.49	0.03	14.79	0.001	0.42	0.55
Entitlement/Grandiosity - Age	-0.04	0.03	-1.33	0.183	-0.10	0.02
Entitlement/Grandiosity - Hours spent using Facebook	0.03	0.03	0.96	0.336	-0.03	0.10
Entitlement/Grandiosity - Gender	0.02	0.03	0.53	0.594	-0.05	0.08
Insufficient self-control/self-discipline - Approval seeking	0.60	0.03	22.89	0.001	0.55	0.65
Insufficient self-control/self-discipline - Pessimism/Negativism	0.67	0.02	28.61	0.001	0.62	0.71
Insufficient self-control/self-discipline - Self-punitiveness	0.65	0.02	27.33	0.001	0.60	0.69
Insufficient self-control/self-discipline - Age	-0.14	0.03	-4.50	0.001	-0.20	-0.08
Insufficient self-control/self-discipline - Hours spent using Facebook	0.07	0.04	2.00	0.045	0.00	0.14
Insufficient self-control/self-discipline - Gender	0.05	0.03	1.70	0.089	-0.01	0.12
Approval seeking - Pessimism/Negativism	0.58	0.03	20.17	0.001	0.53	0.64
Approval seeking - Self-punitiveness	0.53	0.03	17.97	0.001	0.48	0.59
Approval seeking - Age	-0.13	0.03	-4.28	0.001	-0.20	-0.07
Approval seeking - Hours spent using Facebook	0.05	0.03	1.61	0.108	-0.01	0.12
Approval seeking - Gender	0.04	0.03	1.23	0.218	-0.02	0.10
Pessimism/Negativism - Self-punitiveness	0.67	0.02	29.37	0.001	0.62	0.71
Pessimism/Negativism - Age	-0.08	0.03	-2.54	0.011	-0.14	-0.02
Pessimism/Negativism - Hours spent using Facebook	0.03	0.03	1.02	0.307	-0.03	0.10
Pessimism/Negativism - Gender	0.06	0.03	1.85	0.064	0.00	0.12
Self-punitiveness – Age	-0.07	0.03	-2.37	0.018	-0.14	-0.01
Self-punitiveness - Hours spent using Facebook	0.06	0.03	1.76	0.079	-0.01	0.13
Self-punitiveness - Gender	-0.08	0.03	-2.57	0.010	-0.14	-0.02
Age - Hours spent using Facebook	0.00	0.03	0.00	0.998	-0.06	0.06
Age – Gender	-0.11	0.03	-3.40	0.001	-0.17	-0.05
Hours spent using Facebook - Gender	0.04	0.03	1.37	0.171	-0.02	0.11
Intrapersonal stress - External stress	0.61	0.02	26.86	0.001	0.57	0.66
Intrapersonal stress - Emotional tension	0.66	0.02	30.76	0.001	0.62	0.70
External stress - Emotional tension	0.60	0.02	24.99	0.001	0.56	0.65
Emotional deprivation - Abandonment	0.59	0.03	22.07	0.001	0.54	0.65
Emotional deprivation - Mistrust/Abuse	0.77	0.02	50.90	0.001	0.74	0.80
Emotional deprivation - Social isolation/Alienation	0.70	0.02	33.14	0.001	0.66	0.74
Emotional deprivation - Defectiveness/Shame	0.80	0.02	51.56	0.001	0.77	0.83

Emotional deprivation - Failure to achieve	0.70	0.02	35.19	0.001	0.66	0.74
Emotional deprivation - Dependence/Incompetence	0.71	0.02	35.31	0.001	0.67	0.75
Emotional deprivation - Vulnerability to harm or illness	0.65	0.02	27.17	0.001	0.60	0.70
Emotional deprivation – Enmeshment	0.65	0.02	26.39	0.001	0.60	0.69
Emotional deprivation – Subjugation	0.77	0.02	47.07	0.001	0.74	0.80
Emotional deprivation - Self-sacrifice	0.45	0.03	14.37	0.001	0.39	0.51
Emotional deprivation - Emotional inhibition	0.66	0.02	30.21	0.001	0.62	0.71
Emotional deprivation - Unrelenting standards	0.50	0.03	17.34	0.001	0.44	0.55
Emotional deprivation - Entitlement/Grandiosity	0.49	0.03	16.66	0.001	0.44	0.55
Emotional deprivation - Insufficient self-control/self-discipline	0.60	0.02	23.95	0.001	0.55	0.65
Emotional deprivation - Approval seeking	0.47	0.03	15.79	0.001	0.42	0.53
Emotional deprivation - Pessimism/Negativism	0.65	0.02	29.47	0.001	0.61	0.70
Emotional deprivation - Self-punitiveness	0.66	0.02	28.37	0.001	0.61	0.70
Emotional deprivation – Age	-0.03	0.03	-1.03	0.305	-0.09	0.03
Emotional deprivation - Hours spent using Facebook	0.05	0.03	1.61	0.108	-0.01	0.10
Emotional deprivation – Gender	-0.06	0.03	-1.85	0.064	-0.12	0.00
Abandonment - Mistrust/Abuse	0.72	0.02	34.05	0.001	0.68	0.76
Abandonment - Social isolation/Alienation	0.59	0.03	21.58	0.001	0.54	0.65
Abandonment - Defectiveness/Shame	0.66	0.02	28.37	0.001	0.61	0.70
Abandonment - Failure to achieve	0.67	0.02	28.11	0.001	0.63	0.72
Abandonment - Dependence/Incompetence	0.70	0.02	33.74	0.001	0.66	0.74
Abandonment - Vulnerability to harm or illness	0.77	0.02	44.65	0.001	0.73	0.80
Abandonment – Enmeshment	0.58	0.03	21.90	0.001	0.53	0.64
Abandonment – Subjugation	0.73	0.02	40.20	0.001	0.70	0.77
Abandonment - Self-sacrifice	0.53	0.03	18.19	0.001	0.47	0.59
Abandonment - Emotional inhibition	0.58	0.03	20.51	0.001	0.52	0.63
Abandonment - Unrelenting standards	0.61	0.03	24.14	0.001	0.56	0.66
Abandonment - Entitlement/Grandiosity	0.54	0.03	18.67	0.001	0.48	0.59
Abandonment - Insufficient self-control/self-discipline	0.63	0.03	24.63	0.001	0.58	0.68
Abandonment - Approval seeking	0.63	0.02	25.47	0.001	0.58	0.68
Abandonment - Pessimism/Negativism	0.75	0.02	38.15	0.001	0.71	0.78
Abandonment - Self-punitiveness	0.64	0.02	25.48	0.001	0.59	0.68
Abandonment – Age	-0.12	0.03	-3.68	0.001	-0.18	-0.05
Abandonment - Hours spent using Facebook	0.06	0.03	1.69	0.091	-0.01	0.12
Abandonment – Gender	0.07	0.03	2.09	0.036	0.00	0.13
Mistrust/Abuse - Social isolation/Alienation	0.74	0.02	41.95	0.001	0.71	0.78
Mistrust/Abuse - Defectiveness/Shame	0.75	0.02	42.87	0.001	0.72	0.79
Mistrust/Abuse - Failure to achieve	0.71	0.02	34.31	0.001	0.67	0.75
Mistrust/Abuse - Dependence/Incompetence	0.71	0.02	33.04	0.001	0.67	0.75
Mistrust/Abuse - Vulnerability to harm or illness	0.78	0.02	50.90	0.001	0.75	0.81
Mistrust/Abuse – Enmeshment	0.62	0.02	24.81	0.001	0.57	0.67
Mistrust/Abuse – Subjugation	0.79	0.01	54.45	0.001	0.76	0.81
Mistrust/Abuse - Self-sacrifice	0.55	0.03	20.23	0.001	0.50	0.61
Mistrust/Abuse - Emotional inhibition	0.69	0.02	31.95	0.001	0.65	0.73
Mistrust/Abuse - Unrelenting standards	0.65	0.02	26.57	0.001	0.61	0.70

Mistrust/Abuse - Entitlement/Grandiosity	0.59	0.03	21.25	0.001	0.54	0.65
Mistrust/Abuse - Insufficient self-control/self-discipline	0.66	0.02	27.98	0.001	0.62	0.71
Mistrust/Abuse - Approval seeking	0.60	0.03	21.34	0.001	0.54	0.65
Mistrust/Abuse - Pessimism/Negativism	0.82	0.01	61.25	0.001	0.79	0.84
Mistrust/Abuse - Self-punitiveness	0.71	0.02	35.77	0.001	0.67	0.75
Mistrust/Abuse – Age	-0.06	0.03	-2.05	0.040	-0.13	0.00
Mistrust/Abuse - Hours spent using Facebook	0.02	0.03	0.56	0.579	-0.05	0.08
Mistrust/Abuse – Gender	-0.01	0.03	-0.29	0.774	-0.07	0.05
Social isolation/Alienation - Defectiveness/Shame	0.66	0.02	28.92	0.001	0.61	0.70
Social isolation/Alienation - Failure to achieve	0.70	0.02	30.78	0.001	0.66	0.75
Social isolation/Alienation - Dependence/Incompetence	0.65	0.02	28.30	0.001	0.61	0.70
Social isolation/Alienation - Vulnerability to harm or illness	0.61	0.02	24.77	0.001	0.57	0.66
Social isolation/Alienation – Enmeshment	0.53	0.03	19.41	0.001	0.48	0.59
Social isolation/Alienation – Subjugation	0.70	0.02	35.50	0.001	0.67	0.74
Social isolation/Alienation - Self-sacrifice	0.40	0.03	11.89	0.001	0.34	0.47
Social isolation/Alienation - Emotional inhibition	0.76	0.02	44.26	0.001	0.72	0.79
Social isolation/Alienation - Unrelenting standards	0.52	0.03	17.82	0.001	0.47	0.58
Social isolation/Alienation - Entitlement/Grandiosity	0.49	0.03	15.99	0.001	0.43	0.55
Social isolation/Alienation - Insufficient self-control/self-discipline	0.63	0.02	26.38	0.001	0.59	0.68
Social isolation/Alienation - Approval seeking	0.44	0.03	13.27	0.001	0.38	0.51
Social isolation/Alienation - Pessimism/Negativism	0.69	0.02	29.99	0.001	0.64	0.73
Social isolation/Alienation - Self-punitiveness	0.62	0.02	25.71	0.001	0.57	0.66
Social isolation/Alienation – Age	-0.08	0.03	-2.75	0.006	-0.14	-0.02
Social isolation/Alienation - Hours spent using Facebook	-0.03	0.03	-0.98	0.325	-0.09	0.03
Social isolation/Alienation - Gender	0.01	0.03	0.20	0.841	-0.06	0.07
Defectiveness/Shame - Failure to achieve	0.76	0.02	45.33	0.001	0.73	0.79
Defectiveness/Shame - Dependence/Incompetence	0.79	0.02	49.06	0.001	0.76	0.82
Defectiveness/Shame - Vulnerability to harm or illness	0.69	0.02	31.98	0.001	0.65	0.73
Defectiveness/Shame - Enmeshment	0.70	0.02	32.23	0.001	0.66	0.75
Defectiveness/Shame - Subjugation	0.80	0.02	52.06	0.001	0.77	0.83
Defectiveness/Shame - Self-sacrifice	0.36	0.03	10.48	0.001	0.29	0.43
Defectiveness/Shame - Emotional inhibition	0.69	0.02	34.01	0.001	0.65	0.73
Defectiveness/Shame - Unrelenting standards	0.49	0.03	16.24	0.001	0.43	0.55
Defectiveness/Shame - Entitlement/Grandiosity	0.45	0.03	13.60	0.001	0.38	0.51
Defectiveness/Shame - Insufficient self-control/self-discipline	0.65	0.02	28.83	0.001	0.61	0.70
Defectiveness/Shame - Approval seeking	0.49	0.03	16.12	0.001	0.43	0.55
Defectiveness/Shame - Pessimism/Negativism	0.67	0.02	28.76	0.001	0.62	0.71
Defectiveness/Shame - Self-punitiveness	0.77	0.02	50.52	0.001	0.74	0.80
Defectiveness/Shame - Age	-0.11	0.03	-3.64	0.001	-0.17	-0.05
Defectiveness/Shame - Hours spent using Facebook	0.05	0.03	1.61	0.107	-0.01	0.11
Defectiveness/Shame – Gender	-0.07	0.03	-2.22	0.026	-0.13	-0.01
Failure to achieve - Dependence/Incompetence	0.79	0.02	52.52	0.001	0.76	0.82
Failure to achieve - Vulnerability to harm or illness	0.73	0.02	35.72	0.001	0.69	0.77
Failure to achieve – Enmeshment	0.61	0.03	23.18	0.001	0.56	0.66
Failure to achieve – Subjugation	0.78	0.02	44.25	0.001	0.75	0.81

Failure to achieve - Self-sacrifice	0.41	0.03	12.13	0.001	0.35	0.48
Failure to achieve - Emotional inhibition	0.69	0.02	29.35	0.001	0.64	0.73
Failure to achieve - Unrelenting standards	0.51	0.03	16.34	0.001	0.45	0.57
Failure to achieve - Entitlement/Grandiosity	0.45	0.03	13.63	0.001	0.39	0.52
Failure to achieve - Insufficient self-control/self-discipline	0.74	0.02	42.57	0.001	0.71	0.77
Failure to achieve - Approval seeking	0.54	0.03	18.44	0.001	0.48	0.59
Failure to achieve - Pessimism/Negativism	0.74	0.02	36.99	0.001	0.70	0.78
Failure to achieve - Self-punitiveness	0.73	0.02	35.73	0.001	0.69	0.77
Failure to achieve – Age	-0.13	0.03	-4.12	0.001	-0.19	-0.07
Failure to achieve - Hours spent using Facebook	0.06	0.03	1.76	0.078	-0.01	0.12
Failure to achieve – Gender	0.05	0.03	1.48	0.139	-0.02	0.11
Dependence/Incompetence - Vulnerability to harm or illness	0.74	0.02	40.85	0.001	0.70	0.77
Dependence/Incompetence – Enmeshment	0.75	0.02	39.59	0.001	0.71	0.78
Dependence/Incompetence – Subjugation	0.82	0.01	56.43	0.001	0.79	0.85
Dependence/Incompetence - Self-sacrifice	0.41	0.04	11.21	0.001	0.33	0.48
Dependence/Incompetence - Emotional inhibition	0.69	0.02	35.28	0.001	0.65	0.73
Dependence/Incompetence - Unrelenting standards	0.50	0.03	16.21	0.001	0.44	0.57
Dependence/Incompetence - Entitlement/Grandiosity	0.52	0.03	17.59	0.001	0.46	0.58
Dependence/Incompetence - Insufficient self-control/self-discipline	0.74	0.02	39.71	0.001	0.70	0.78
Dependence/Incompetence - Approval seeking	0.56	0.03	20.75	0.001	0.51	0.62
Dependence/Incompetence - Pessimism/Negativism	0.69	0.02	33.47	0.001	0.65	0.73
Dependence/Incompetence - Self-punitiveness	0.72	0.02	34.31	0.001	0.68	0.76
Dependence/Incompetence – Age	-0.12	0.03	-4.00	0.001	-0.19	-0.06
Dependence/Incompetence - Hours spent using Facebook	0.08	0.03	2.42	0.015	0.02	0.15
Dependence/Incompetence – Gender	0.01	0.03	0.28	0.776	-0.05	0.07
Vulnerability to harm or illness - Enmeshment	0.64	0.02	25.85	0.001	0.59	0.69
Vulnerability to harm or illness - Subjugation	0.75	0.02	42.09	0.001	0.71	0.78
Vulnerability to harm or illness - Self-sacrifice	0.50	0.03	16.88	0.001	0.45	0.56
Vulnerability to harm or illness - Emotional inhibition	0.62	0.03	24.86	0.001	0.57	0.67
Vulnerability to harm or illness - Unrelenting standards	0.61	0.03	23.63	0.001	0.56	0.66
Vulnerability to harm or illness - Entitlement/Grandiosity	0.56	0.03	20.03	0.001	0.51	0.62
Vulnerability to harm or illness - Insufficient self-control/self-discipline	0.66	0.02	29.81	0.001	0.62	0.71
Vulnerability to harm or illness - Approval seeking	0.58	0.03	21.55	0.001	0.53	0.63
Vulnerability to harm or illness - Pessimism/Negativism	0.84	0.01	75.34	0.001	0.82	0.86
Vulnerability to harm or illness - Self-punitiveness	0.68	0.02	29.66	0.001	0.63	0.72
Vulnerability to harm or illness - Age	-0.06	0.03	-1.75	0.081	-0.12	0.01
Vulnerability to harm or illness - Hours spent using Facebook	0.07	0.03	2.17	0.030	0.01	0.14
Vulnerability to harm or illness - Gender	0.04	0.03	1.40	0.162	-0.02	0.11
Enmeshment - Subjugation	0.70	0.02	33.20	0.001	0.66	0.74
Enmeshment - Self-sacrifice	0.42	0.03	12.92	0.001	0.36	0.49
Enmeshment - Emotional inhibition	0.57	0.03	22.25	0.001	0.52	0.62
Enmeshment - Unrelenting standards	0.49	0.03	16.34	0.001	0.43	0.55
Enmeshment - Entitlement/Grandiosity	0.55	0.03	18.94	0.001	0.49	0.61
Enmeshment - Insufficient self-control/self-discipline	0.59	0.03	20.73	0.001	0.53	0.64

Enmeshment - Approval seeking	0.48	0.03	16.15	0.001	0.42	0.54
Enmeshment - Pessimism/Negativism	0.54	0.03	18.46	0.001	0.48	0.59
Enmeshment - Self-punitiveness	0.66	0.02	27.63	0.001	0.61	0.71
Enmeshment - Age	-0.06	0.03	-1.92	0.055	-0.12	0.00
Enmeshment - Hours spent using Facebook	0.12	0.04	3.33	0.001	0.05	0.19
Enmeshment - Gender	-0.05	0.03	-1.56	0.118	-0.11	0.01
Subjugation - Self-sacrifice	0.50	0.03	16.39	0.001	0.44	0.56
Subjugation - Emotional inhibition	0.73	0.02	39.18	0.001	0.70	0.77
Subjugation - Unrelenting standards	0.62	0.03	24.63	0.001	0.57	0.67
Subjugation - Entitlement/Grandiosity	0.55	0.03	18.72	0.001	0.49	0.60
Subjugation - Insufficient self-control/self-discipline	0.71	0.02	32.79	0.001	0.67	0.75
Subjugation - Approval seeking	0.60	0.03	22.23	0.001	0.54	0.65
Subjugation - Pessimism/Negativism	0.76	0.02	47.63	0.001	0.73	0.79
Subjugation - Self-punitiveness	0.74	0.02	38.99	0.001	0.71	0.78
Subjugation - Age	-0.09	0.03	-2.72	0.006	-0.15	-0.02
Subjugation - Hours spent using Facebook	0.02	0.03	0.53	0.597	-0.04	0.08
Subjugation - Gender	0.00	0.03	-0.07	0.945	-0.06	0.06
Self-sacrifice - Emotional inhibition	0.31	0.04	8.09	0.001	0.23	0.38
Self-sacrifice - Unrelenting standards	0.59	0.03	20.66	0.001	0.53	0.64
Self-sacrifice - Entitlement/Grandiosity	0.49	0.03	14.81	0.001	0.43	0.56
Self-sacrifice - Insufficient self-control/self-discipline	0.39	0.04	10.90	0.001	0.32	0.46
Self-sacrifice - Approval seeking	0.45	0.03	13.22	0.001	0.38	0.52
Self-sacrifice - Pessimism/Negativism	0.52	0.03	17.27	0.001	0.46	0.58
Self-sacrifice - Self-punitiveness	0.46	0.03	13.59	0.001	0.39	0.53
Self-sacrifice - Age	0.04	0.03	1.12	0.264	-0.03	0.10
Self-sacrifice - Hours spent using Facebook	0.05	0.03	1.78	0.074	-0.01	0.11
Self-sacrifice - Gender	0.11	0.03	3.60	0.001	0.05	0.17
Emotional inhibition - Unrelenting standards	0.52	0.03	17.22	0.001	0.46	0.57
Emotional inhibition - Entitlement/Grandiosity	0.47	0.03	14.17	0.001	0.40	0.53
Emotional inhibition - Insufficient self-control/self-discipline	0.65	0.02	26.87	0.001	0.61	0.70
Emotional inhibition - Approval seeking	0.46	0.03	14.53	0.001	0.40	0.52
Emotional inhibition - Pessimism/Negativism	0.67	0.02	29.54	0.001	0.63	0.72
Emotional inhibition - Self-punitiveness	0.62	0.03	23.82	0.001	0.56	0.67
Emotional inhibition - Age	-0.07	0.03	-2.32	0.020	-0.13	-0.01
Emotional inhibition - Hours spent using Facebook	0.00	0.04	0.03	0.974	-0.07	0.07
Emotional inhibition - Gender	-0.03	0.03	-0.97	0.332	-0.09	0.03
Unrelenting standards - Entitlement/Grandiosity	0.59	0.03	21.66	0.001	0.54	0.65
Unrelenting standards - Insufficient self-control/self-discipline	0.50	0.03	14.56	0.001	0.44	0.57
Unrelenting standards - Approval seeking	0.65	0.02	26.37	0.001	0.60	0.70
Unrelenting standards - Pessimism/Negativism	0.65	0.03	24.74	0.001	0.60	0.70
Unrelenting standards - Self-punitiveness	0.65	0.02	27.32	0.001	0.60	0.70
Unrelenting standards - Age	-0.05	0.03	-1.51	0.131	-0.11	0.01
Unrelenting standards - Hours spent using Facebook	-0.03	0.03	-0.83	0.405	-0.09	0.04
Unrelenting standards - Gender	-0.01	0.03	-0.29	0.768	-0.07	0.05
Entitlement/Grandiosity - Insufficient self-control/self-discipline	0.56	0.03	19.55	0.001	0.51	0.62

Entitlement/Grandiosity - Approval seeking	0.64	0.02	26.71	0.001	0.59	0.68
Entitlement/Grandiosity - Pessimism/Negativism	0.51	0.03	17.21	0.001	0.46	0.57
Entitlement/Grandiosity - Self-punitiveness	0.49	0.03	14.79	0.001	0.42	0.55
Entitlement/Grandiosity - Age	-0.04	0.03	-1.33	0.183	-0.10	0.02
Entitlement/Grandiosity - Hours spent using Facebook	0.03	0.03	0.96	0.336	-0.03	0.10
Entitlement/Grandiosity - Gender	0.02	0.03	0.53	0.594	-0.05	0.08
Insufficient self-control/self-discipline - Approval seeking	0.60	0.03	22.89	0.001	0.55	0.65
Insufficient self-control/self-discipline - Pessimism/Negativism	0.67	0.02	28.61	0.001	0.62	0.71
Insufficient self-control/self-discipline - Self-punitiveness	0.65	0.02	27.33	0.001	0.60	0.69
Insufficient self-control/self-discipline - Age	-0.14	0.03	-4.50	0.001	-0.20	-0.08
Insufficient self-control/self-discipline - Hours spent using Facebook	0.07	0.04	2.00	0.045	0.00	0.14
Insufficient self-control/self-discipline - Gender	0.05	0.03	1.70	0.089	-0.01	0.12
Approval seeking - Pessimism/Negativism	0.58	0.03	20.17	0.001	0.53	0.64
Approval seeking - Self-punitiveness	0.53	0.03	17.97	0.001	0.48	0.59
Approval seeking - Age	-0.13	0.03	-4.28	0.001	-0.20	-0.07
Approval seeking - Hours spent using Facebook	0.05	0.03	1.61	0.108	-0.01	0.12
Approval seeking - Gender	0.04	0.03	1.23	0.218	-0.02	0.10
Pessimism/Negativism - Self-punitiveness	0.67	0.02	29.37	0.001	0.62	0.71
Pessimism/Negativism - Age	-0.08	0.03	-2.54	0.011	-0.14	-0.02
Pessimism/Negativism - Hours spent using Facebook	0.03	0.03	1.02	0.307	-0.03	0.10
Pessimism/Negativism - Gender	0.06	0.03	1.85	0.064	0.00	0.12
Self-punitiveness - Age	-0.07	0.03	-2.37	0.018	-0.14	-0.01
Self-punitiveness - Hours spent using Facebook	0.06	0.03	1.76	0.079	-0.01	0.13
Self-punitiveness - Gender	-0.08	0.03	-2.57	0.010	-0.14	-0.02
Age - Hours spent using Facebook	0.00	0.03	0.00	0.998	-0.06	0.06
Age – Gender	-0.11	0.03	-3.40	0.001	-0.17	-0.05
Hours spent using Facebook - Gender	0.04	0.03	1.37	0.171	-0.02	0.11
