# Supplementary material for: The Relationship between Early Maladaptive Schemas and Problematic Facebook Use: The Indirect Effects of Perceived Stress
Source: Int J Environ Res Public Health. 2023 Feb 8;20(4):2969. doi: 10.3390/ijerph20042969 (PMC9957475; doi:10.3390/ijerph20042969)
Supplement: Supplementary file 1 [file ijerph-20-02969-s001.zip › ijerph-2073240-supplementary/Table S3.pdf]

Table S3. Standardized indirect effects exerted by emotional tension and intrapersonal stress in relationship between EMSs and PFU with 95% confidence intervals (CIs) among Facebook users (N = 993)

Pathways	Point estimates	Standard error	z	*p*	95% CIs	
					Lower	Upper
Emotional deprivation → Emotional tension → PFU	0.005	0.006	0.74	0.460	-0.005	0.019
Abandonment→ Emotional tension → PFU	0.013	0.009	1.41	0.158	-0.001	0.033
Mistrust/Abuse→ Emotional tension → PFU	-0.001	0.007	-0.06	0.955	-0.015	0.013
Social isolation/Alienation→ Emotional tension → PFU	0.005	0.006	0.91	0.360	-0.004	0.019
Defectiveness/Shame→ Emotional tension → PFU	-0.004	0.007	-0.60	0.547	-0.020	0.007
Failure to achieve→ Emotional tension → PFU	0.004	0.007	0.61	0.542	-0.007	0.019
Dependence/Incompetence → Emotional tension → PFU	0.009	0.008	1.13	0.257	-0.003	0.029
Vulnerability to harm or illness→ Emotional tension → PFU	0.011	0.009	1.25	0.213	-0.002	0.032
Enmeshment→ Emotional tension → PFU	-0.007	0.006	-1.11	0.267	-0.021	0.002
Subjugation→ Emotional tension → PFU	0.006	0.007	0.81	0.419	-0.006	0.024
Self-sacrifice→ Emotional tension → PFU	-0.003	0.004	-0.64	0.524	-0.012	0.004
Emotional inhibition→ Emotional tension → PFU	-0.003	0.005	-0.53	0.594	-0.014	0.006
Unrelenting standards→ Emotional tension → PFU	0.003	0.005	0.63	0.527	-0.006	-0.016
Entitlement/Grandiosity→ Emotional tension → PFU	-0.016	0.010	-1.61	0.108	-0.037	0.002
Insufficient self-control/self-discipline→ Emotional tension → PFU	0.015	0.010	1.52	0.129	-0.002	0.036
Approval seeking→ Emotional tension → PFU	0.002	0.005	0.37	0.709	-0.007	0.012
Pessimism/Negativism→ Emotional tension → PFU	0.017	0.012	1.47	0.142	-0.002	0.043
Self-punitiveness→ Emotional tension → PFU	-0.016	0.011	-1.55	0.121	-0.040	0.002
Emotional deprivation → Intrapsychic stress → PFU	0.008	0.008	1.01	0.311	-0.006	0.027
Abandonment → Intrapsychic stress → PFU	0.006	0.007	0.94	0.345	-0.005	0.022
Mistrust/Abuse → Intrapsychic stress → PFU	0.005	0.007	0.80	0.425	-0.005	0.021
Social isolation/Alienation → Intrapsychic stress → PFU	0.005	0.006	0.83	0.409	-0.004	0.018
Defectiveness/Shame → Intrapsychic stress → PFU	-0.003	0.005	-0.51	0.611	-0.015	0.006
Failure to achieve → Intrapsychic stress → PFU	0.014	0.013	1.10	0.272	-0.010	0.041
Dependence/Incompetence → Intrapsychic stress → PFU	-0.001	0.004	-0.05	0.957	-0.010	0.009
Vulnerability to harm or illness → Intrapsychic stress → PFU	0.012	0.011	1.06	0.289	-0.008	0.036
Enmeshment → Intrapsychic stress → PFU	-0.002	0.004	-0.56	0.579	-0.012	0.004
Subjugation → Intrapsychic stress → PFU	0.009	0.009	0.98	0.325	-0.006	0.029
Self-sacrifice → Intrapsychic stress → PFU	-0.003	0.004	-0.83	0.408	-0.013	0.003
Emotional inhibition → Intrapsychic stress → PFU	-0.002	0.004	-0.55	0.580	-0.012	0.004
Unrelenting standards → Intrapsychic stress → PFU	0.001	0.004	0.33	0.745	-0.006	0.010
Entitlement/Grandiosity → Intrapsychic stress → PFU	-0.012	0.011	-1.12	0.262	-0.035	0.008
Insufficient self-control/self-discipline → Intrapsychic stress → PFU	0.005	0.005	0.85	0.393	-0.004	0.018
Approval seeking → Intrapsychic stress → PFU	-0.001	0.003	-0.34	0.732	-0.009	0.005
Pessimism/Negativism → Intrapsychic stress → PFU	0.004	0.006	0.70	0.487	-0.005	0.018
Self-punitiveness → Intrapsychic stress → PFU	-0.009	0.009	-1.06	0.290	-0.028	0.006

Note: PFU = problematic Facebook use; Emboldened variables = statistically significant
